# The Preparation of Hypoallergenic Goat Milk Protein Hydrolysates via Targeted Hydrolysis of Cross-Reactive Linear Epitopes Between Cow and Goat Milk

**DOI:** 10.3390/ijms27156603

**Published:** 2026-07-24

**Authors:** Fengyi Wang, Wenxuan Zhao, Yanjun Cong

**Affiliations:** Beijing Engineering and Technology Research Center of Food Additives, College of Food and Health, Beijing Technology and Business University, Beijing 100048, China; wangfengyi03@163.com (F.W.); 18634443697@163.com (W.Z.)

**Keywords:** goat milk protein, cow milk protein, cross reactivity, linear epitope, enzymatic hydrolysis, allergenicity

## Abstract

Cow’s milk allergy (CMA) is the most common food allergy in infants. Goat milk exhibits relatively low allergenicity and is widely regarded as a potential substitute for cow milk. However, the high sequence homology between cow and goat milk proteins may trigger cross-reactivity, significantly restricting the practical application of goat milk. This study aims to disrupt cross-linear epitopes of cow and goat milk proteins through enzymatic hydrolysis. We prepare hypoallergenic goat milk protein hydrolysates and systematically evaluate their desensitization effects. Bioinformatics methods were employed to predict the B-cell linear epitopes of six major allergens (α_S1_-casein, α_S2_-casein, β-casein, κ-casein, α-lactalbumin, and β-lactoglobulin) from cow, goat, and sheep milk, and sequence homology was analyzed through protein-protein Basic Local Alignment Search Tool (BLASTP). The results showed that the sequence similarity of homologous allergens among the three milk sources all exceeded 30%. Subsequently, six proteases (protamex, alcalase, pepsin, trypsin, papain and bromelain) were used to hydrolyze whole goat milk protein. Indirect enzyme-linked immunosorbent assay (ELISA) results indicated that all six hydrolysates significantly reduced immunoreactivity with antibodies against the five major cow’s milk allergens. Among them, alcalase exhibited significant efficacy against all five allergens; papain and protamex were particularly effective against β-casein; trypsin showed pronounced efficacy against α-lactalbumin and β-lactoglobulin; and bromelain and pepsin also significantly reduced immunoreactivity against certain allergens. Mass spectrometry revealed the peptide composition and epitope coverage of the hydrolysates: bromelain yielded two overlapping peptides (FAWPQY, LKDLKDY), protamex one (LAMAAS), Alcalase three (PPQSVLS, IPIQYVLS, LPYPYY), trypsin two (YLGYLEQL, AMAASDISLL), papain three (NEINQFYQK, FQSEEQQQTEDELQDK, AMAASDISL); and no matching peptide was detected for pepsin. These results confirmed at the molecular level that enzymatic hydrolysis can effectively disrupt cross-reactive epitopes.

## 1. Introduction

As an indispensable component of the human diet, dairy products serve as a vital source of high-quality protein, calcium, and various vitamins and minerals [1]. Cow milk and goat milk are the two largest milk sources worldwide [2] and represent the two most commercially significant dairy categories, playing a crucial role in human nutrition and health. Cow milk-based infant formula is widely recognized as an ideal breast milk substitute or complementary food for infants, owing to its high digestibility and rich nutritional profile. However, over 20 proteins in cow milk are potential allergens in humans, and the prevalence of cow’s milk allergy (CMA) is rising, with more than 8% of children under 3 years of age affected [3]. CMA is an aberrant immune response directed against milk proteins, arising from the interaction between one or more milk proteins and the immune system. It represents the most common type of food allergy during infancy and early childhood [4] and may elicit clinical symptoms affecting multiple organ systems, ranging from the skin and gastrointestinal tract to the respiratory system, thereby severely compromising the quality of life and growth and development of affected children. In addition to allergies, lactose intolerance (LI) constitutes another common physiological disorder affecting infant health. Therefore, there is a growing need for a safe, effective, and nutritionally complete alternative milk source to meet the dietary requirements of special populations, particularly infants, individuals with CMA, and those with lactose intolerance [5].

Goat milk exhibits a relatively high protein content (ranging from 3.4% to 6.2%) and superior protein quality compared with cow milk (3.2%). Goat milk proteins are richer in essential amino acids, such as lysine, isoleucine, and threonine [6]. Compared with cow milk, goat milk has lower levels of α_S1_-casein, α-lactalbumin, and β-lactoglobulin, while its content of β-casein and α_S2_-casein are relatively higher [7,8,9]. Studies have found that α_S1_-casein and β-lactoglobulin are the major proteins responsible for milk protein allergy, and their lower abundance in goat milk is the primary reason why goat milk can reduce the allergenicity of cow’s milk [10]. It has also been reported that β-casein from both cow and goat milk induces high-level secretion of TNF-α, whereas α_S2_-casein induces IL-1β secretion [11,12,13], both of which are Th1-type cytokines. Given that CMA is predominantly a Th2-type immune response, it is hypothesized that the lower allergenicity of goat milk may be partly attributable to a shift from the Th2-type response toward a Th1-type response, and partly to differences in antigenic surface epitopes that confer distinct sensitization profiles [14].

Furthermore, compared with cow milk, goat milk has a lower lactose content (ranging from 4.1% to 4.9%) and smaller lactose micelles. The α-lactose to β-lactose ratio is 60:40 in goat milk, versus 40:60 in cow milk, which facilitates better digestion and absorption [15], making goat milk suitable for individuals with lactose intolerance.

Although goat milk is widely regarded as a potential substitute for CMA patients due to its low allergenicity and easy digestibility, its universal applicability is significantly limited by the increasingly recognized issue of cross-reactivity [16,17,18]. Immunological studies indicate that the amino acid sequence homology of αs1-casein between cow and goat milk is as high as 89%, and although their antigenic epitopes differ, the number of epitopes is comparable, with overlapping epitope regions [19], thereby leading to Immunoglobulin E (IgE)-mediated allergic reactions to goat milk in CMA children. Current evidence has unequivocally identified α-casein, β-casein, and β-lactoglobulin as the main contributors to cross-reactivity, with the fundamental cause being high sequence similarity but distinct epitope distributions among these proteins [20]. The existence of such cross-reactivity raises concerns regarding the safety of goat milk as an alternative, especially given the current lack of precise screening tools for high-risk populations and effective allergen modulation techniques.

In recent years, the application of liquid chromatography–tandem mass spectrometry has advanced epitope research, allowing researchers to identify peptide composition in enzymatic hydrolysates and thereby deduce whether key allergenic epitopes have been disrupted, thus providing direct evidence for evaluating the efficacy of different proteases in cleaving epitopes [9]. However, most existing studies remain at the predictive stage, lacking a systematic comparison of the epitope-cleavage efficiency among different proteases.

In this study, bioinformatics approaches were employed to predict the B-cell linear epitopes of major allergens from cow, goat, and sheep milk. Sequence homology among the three species was compared via protein-protein Basic Local Alignment Search Tool (BLASTP) analysis to elucidate the molecular basis of cross-allergenicity [21,22]. Subsequently, six proteases with distinct cleavage characteristics were selected to hydrolyze whole goat milk proteins. The hydrolysates were evaluated by sodium dodecyl sulfate-polyacrylamide gel electrophoresis (SDS-PAGE), degree of hydrolysis (DH) determination, and indirect enzyme-linked immunosorbent assay (ELISA) for immunoreactivity assessment, to screen for proteases that most effectively reduced cross reactivity between goat and cow milk proteins. Finally, label-free quantitative proteomic analysis was performed on the enzymatic hydrolysates using nano-liquid chromatography coupled with tandem high-resolution mass spectrometry (nanoLC-Thermo QE Plus). Through peptide identification, the cleavage sites and peptide composition features of each protease were characterized, thereby elucidating the molecular mechanism by which enzymatic hydrolysis reduces cross-reactivity.

## 2. Results

### 2.1. Prediction Results of Sequence Similarities Among Cross-Reactive Allergens from Cow, Goat, and Sheep Milk

#### 2.1.1. Prediction Results of Sequence Similarities Between Cow and Goat Milk Allergens

Pairwise alignments of amino acid sequences of homologous allergens from cow and goat milk were performed using the BLASTP algorithm on the NCBI platform. As shown in Table 1, the sequence similarities of α_S1_-casein, α_S2_-casein, β-casein, κ-casein, α-lactalbumin, and β-lactoglobulin between cow and goat milk all exceeded 30%. Amino acid residues differing between cow and goat milk proteins have been highlighted.

For α_S1_-casein, between cow and goat milk, five similar sequence segments were identified: cow AA53–71 vs. goat AA53–71; cow AA117–196 vs. goat AA117–196; cow AA101–119 vs. goat AA79–98; cow AA125–134 vs. goat AA85–94; and cow AA29–47 vs. goat AA29–47.

For α_S2_-casein, between cow and goat milk, eight similar segments were found: cow AA94–113 vs. goat AA95–114; cow AA179–196 vs. goat AA98–113; cow AA140–159 vs. goat AA141–160; cow AA173–192 vs. goat AA174–193; cow AA97–107 vs. goat AA180–190; cow AA200–219 vs. goat AA201–220; cow AA124–133 vs. goat AA209–218; and cow AA15–29 vs. goat AA15–29.

For β-casein, between cow and goat milk, four similar segments were identified: cow AA193–212 vs. goat AA47–66; cow AA314–332 vs. goat AA167–186; cow AA165–176 vs. goat AA19–30; and cow AA259–278 vs. goat AA113–132.

For κ-casein, between cow and goat milk, three similar segments were found: cow AA39–58 vs. goat AA39–58; cow AA135–152 vs. goat AA135–152; and cow AA74–93 vs. goat AA74–93.

For α-lactalbumin, between cow and goat milk, two similar segments were identified: cow AA113–132 vs. goat AA113–132 and cow AA11–30 vs. goat AA11–30.

For β-lactoglobulin between, cow and goat milk, five similar segments were found: cow AA36–55 vs. goat AA38–57; cow AA103–122 vs. goat AA105–124; cow AA12–30 vs. goat AA14–32; cow AA129–148 vs. goat AA131–150; and cow AA159–178 vs. goat AA161–180.

#### 2.1.2. Prediction Results of Sequence Similarities Between Cow and Sheep Milk Allergens

Pairwise alignments of amino acid sequences of homologous allergens from cow and sheep milk were performed using the BLASTP algorithm on the NCBI platform. As shown in Table 2, the sequence similarities of α_S1_-casein, α_S2_-casein, β-casein, κ-casein, α-lactalbumin, and β-lactoglobulin between cow and sheep milk all exceeded 30%. Amino acid residues differing between cow and sheep milk proteins have been highlighted.

For α_S1_-casein, between cow and sheep milk, five similar segments were identified: cow AA117–196 vs. sheep AA169–188; cow AA101–119 vs. sheep AA101–119; cow AA53–71 vs. sheep AA53–71; cow AA79–98 vs. sheep AA79–98; and cow AA125–134 vs. sheep AA85–94.

For α_S2_-casein, between cow and sheep milk, nine similar segments were found: cow AA97–116 vs. sheep AA98–117; cow AA179–200 vs. sheep AA98–117; cow AA45–64 vs. sheep AA46–65; cow AA140–159 vs. sheep AA141–160; cow AA14–32 vs. sheep AA14–33; cow AA173–192 vs. sheep AA174–193; cow AA97–107 vs. sheep AA180–190; cow AA200–218 vs. sheep AA201–219; and cow AA120–133 vs. sheep AA201–217.

For β-casein, between cow and sheep milk, four similar segments were identified: cow AA47–66 vs. sheep AA46–65; cow AA169–188 vs. sheep AA168–187; cow AA19–30 vs. sheep AA18–29; and cow AA113–132 vs. sheep AA112–131.

For κ-casein, between cow and sheep milk, four similar segments were found: cow AA39–58 vs. sheep AA39–58; cow AA135–152 vs. sheep AA135–152; cow AA168–186 vs. sheep AA170–188; and cow AA74–93 vs. sheep AA74–93.

For α-lactalbumin, between cow and sheep milk, two similar segments were identified: cow AA113–131 vs. sheep AA113–131 and cow AA11–30 vs. sheep AA11–30.

For β-lactoglobulin, between cow and sheep milk, four similar segments were found: cow AA103–122 vs. sheep AA105–124; cow AA36–55 vs. sheep AA38–57; cow AA12–30 vs. sheep AA14–32; and cow AA129–140 vs. sheep AA131–142.

### 2.2. SDS-PAGE Profiles of Whole Goat Milk Protein and Six Enzymatic Hydrolysates

#### 2.2.1. SDS-PAGE Profile of Whole Goat Milk Protein

**Figure 1:**
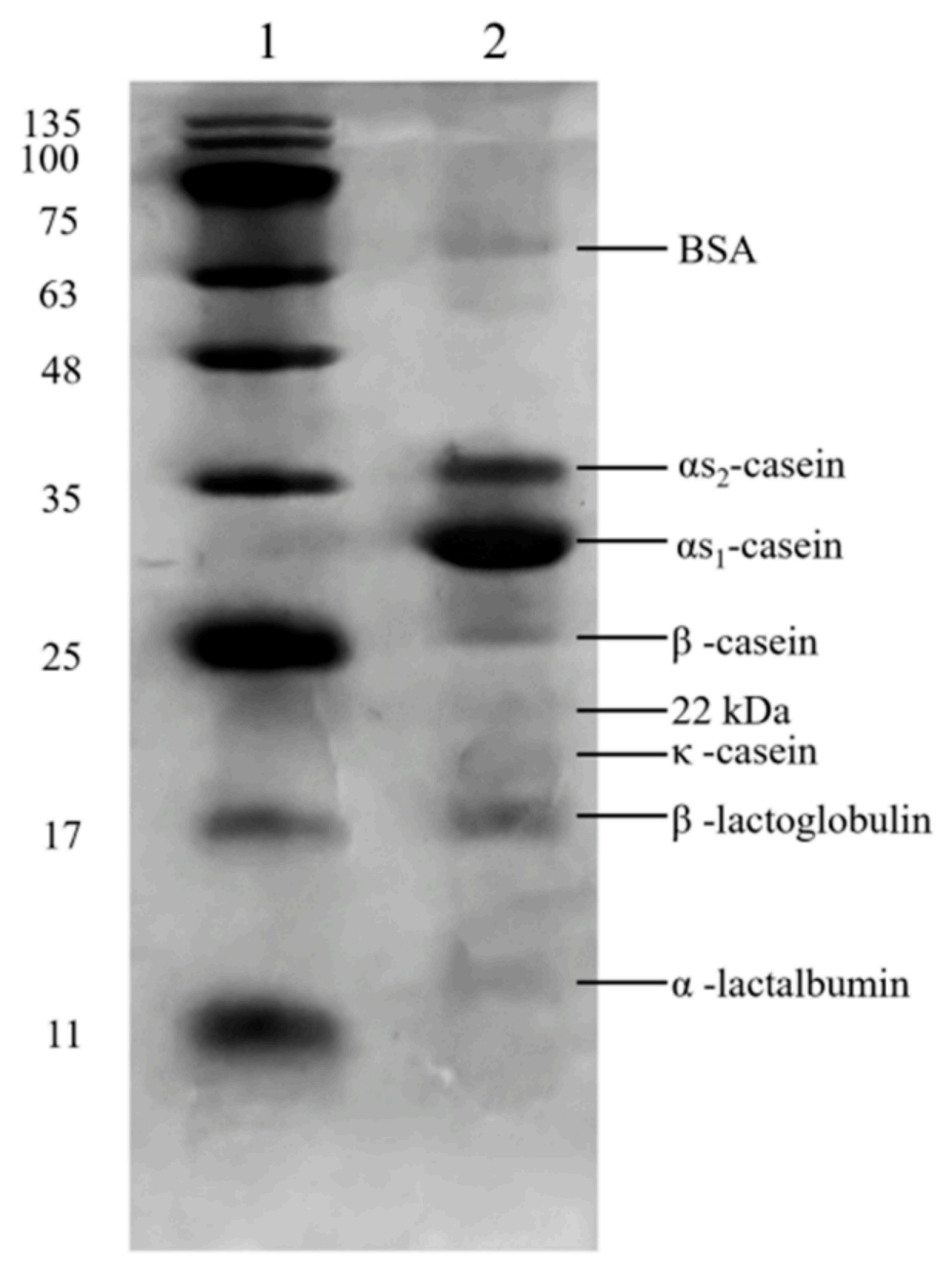
SDS-PAGE profiles of whole goat milk protein. Lane 1: marker. Lane 2: whole goat milk protein.

The SDS-PAGE profile of whole goat milk protein is shown in Figure 1. The bands from top to bottom corresponded to 66 kDa (bovine serum albumin, BSA), 35 kDa (α_S2_-casein), 29 kDa (α_S1_-casein), 25 kDa (β-casein), 19 kDa (κ-casein), 17 kDa (β-lactoglobulin), and 13 kDa (α-lactalbumin).

Besides the major bands, a faint additional band was observed at approximately 22 kDa, which likely represents β-casein isoforms or post-translationally modified forms or a low-abundance whey protein or a minor casein component that co-migrates in this region; its identity was not pursued further as it falls outside the scope of the major allergens targeted in this study.

#### 2.2.2. SDS-PAGE Profiles of the Six Enzymatic Hydrolysates

**Figure 2:**
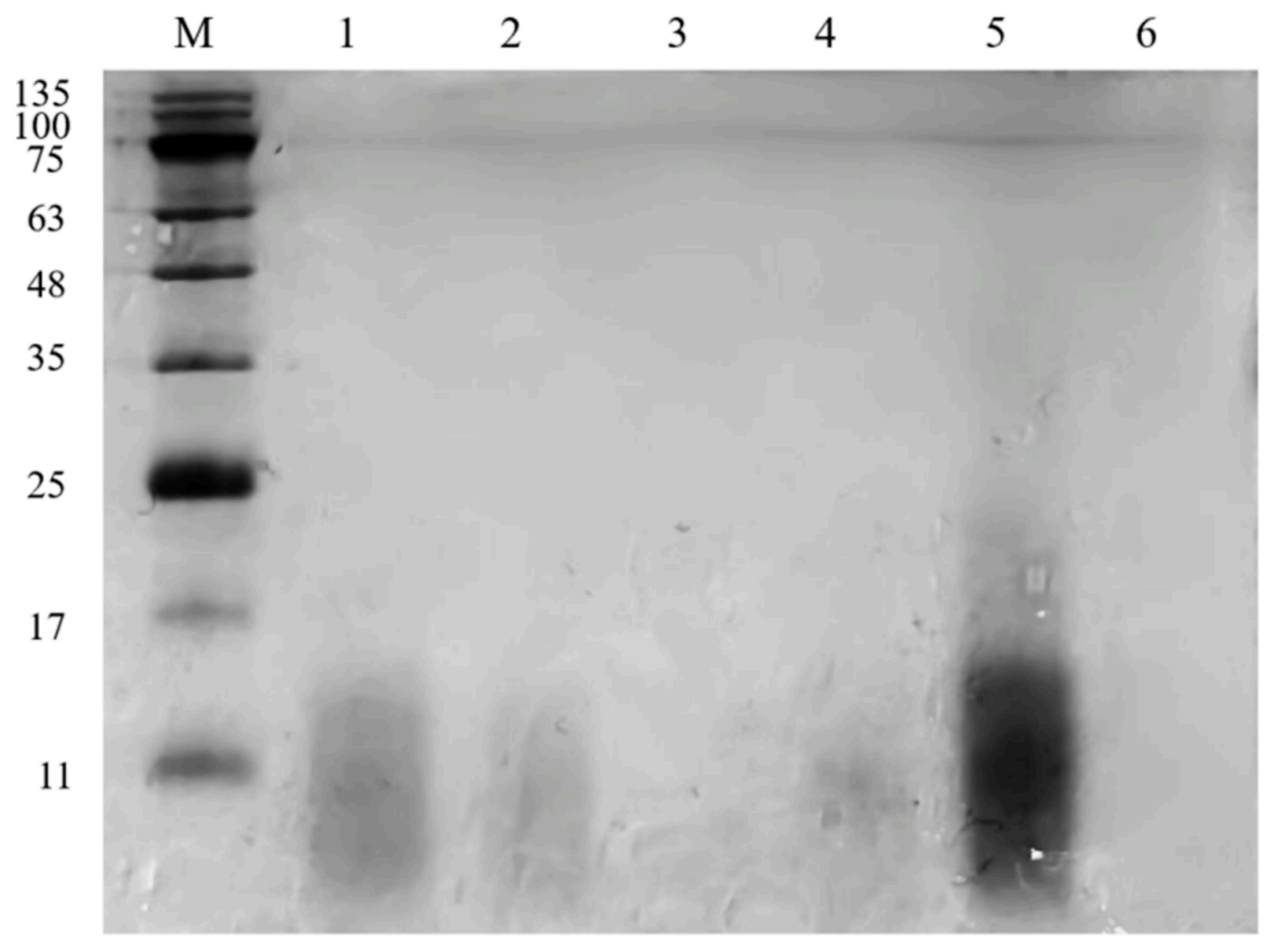
SDS-PAGE profiles of six proteolytic hydrolysates. Lane M: marker; Lane 1: bromelain hydrolysate; Lane 2: papain hydrolysate; Lane 3: alkaline protease hydrolysate; Lane 4: protamex hydrolysate; Lane 5: pepsin hydrolysate; and Lane 6: trypsin hydrolysate.

The SDS-PAGE profiles of the six hydrolysates are shown in Figure 2. Lane M contained the protein molecular weight marker; lanes 1–6 corresponded to the hydrolysates obtained with bromelain, papain, alkaline protease, protamex, pepsin, and trypsin, respectively. As shown in the profiles, the hydrolysates from trypsin (lane 6) and alkaline protease (lane 3) showed almost no visible protein bands, with only weak smearing in the low molecular weight region. The hydrolysates from papain (lane 2) and protamex (lane 4) exhibited smearing below approximately 17 kDa, although a small number of residual bands around 25–35 kDa remained visible. In contrast, the hydrolysates from bromelain (Lane 1) and pepsin (Lane 5) still displayed numerous distinct protein bands, mainly in the 17–48 kDa range.

### 2.3. Determination of the Degree of Hydrolysis

**Figure 3:**
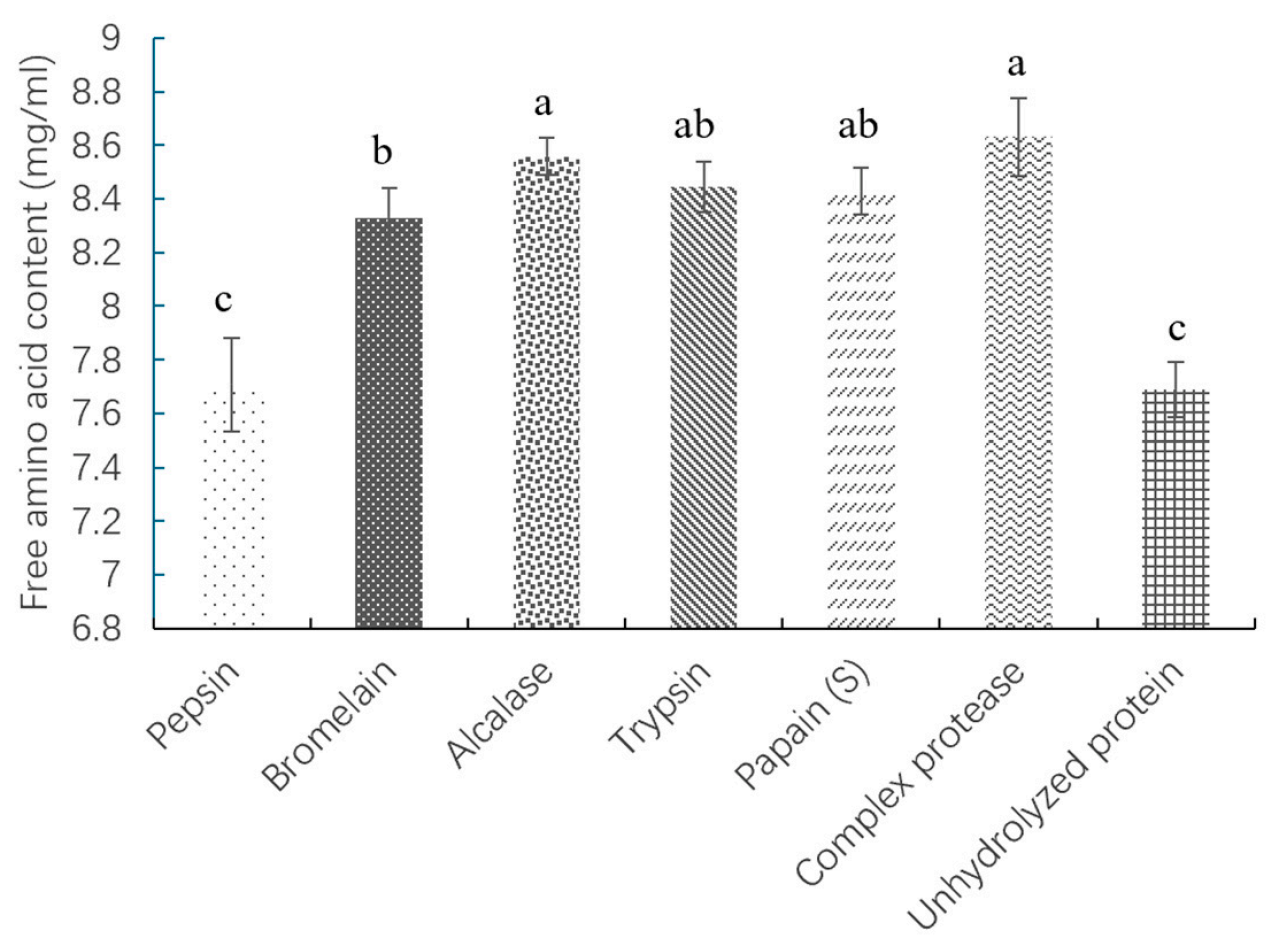
Free amino acid content of the six enzymatic hydrolysates with significance analysis. Different letters represent significance analysis.

The contents of 20 amino acids in whole goat milk protein were determined using an amino acid analyzer, and the results are presented in Table 3. The total amino group content in whole goat milk protein was calculated to be 20.9678 mg/mL. The OD values of unhydrolyzed whole goat milk protein measured by the OPA method were recorded (Table 4), and the free NH_2_ concentration was calculated as 7.689 mg/mL. The free amino acid contents of the six hydrolysates were shown in Figure 3. Compared with the unhydrolyzed original sample, all hydrolysates except the pepsin group exhibited a significant increase in free amino acid content (*p* < 0.05). The measured free amino acid concentrations of the six hydrolysates were as follows: pepsin (7.71 ± 0.174 mg/mL), bromelain (8.33 ± 0.108 mg/mL), alkaline protease (8.56 ± 0.071 mg/mL), trypsin (8.45 ± 0.093 mg/mL), papain (8.43 ± 0.087 mg/mL), and protamex (8.63 ± 0.145 mg/mL).

**Figure 4:**
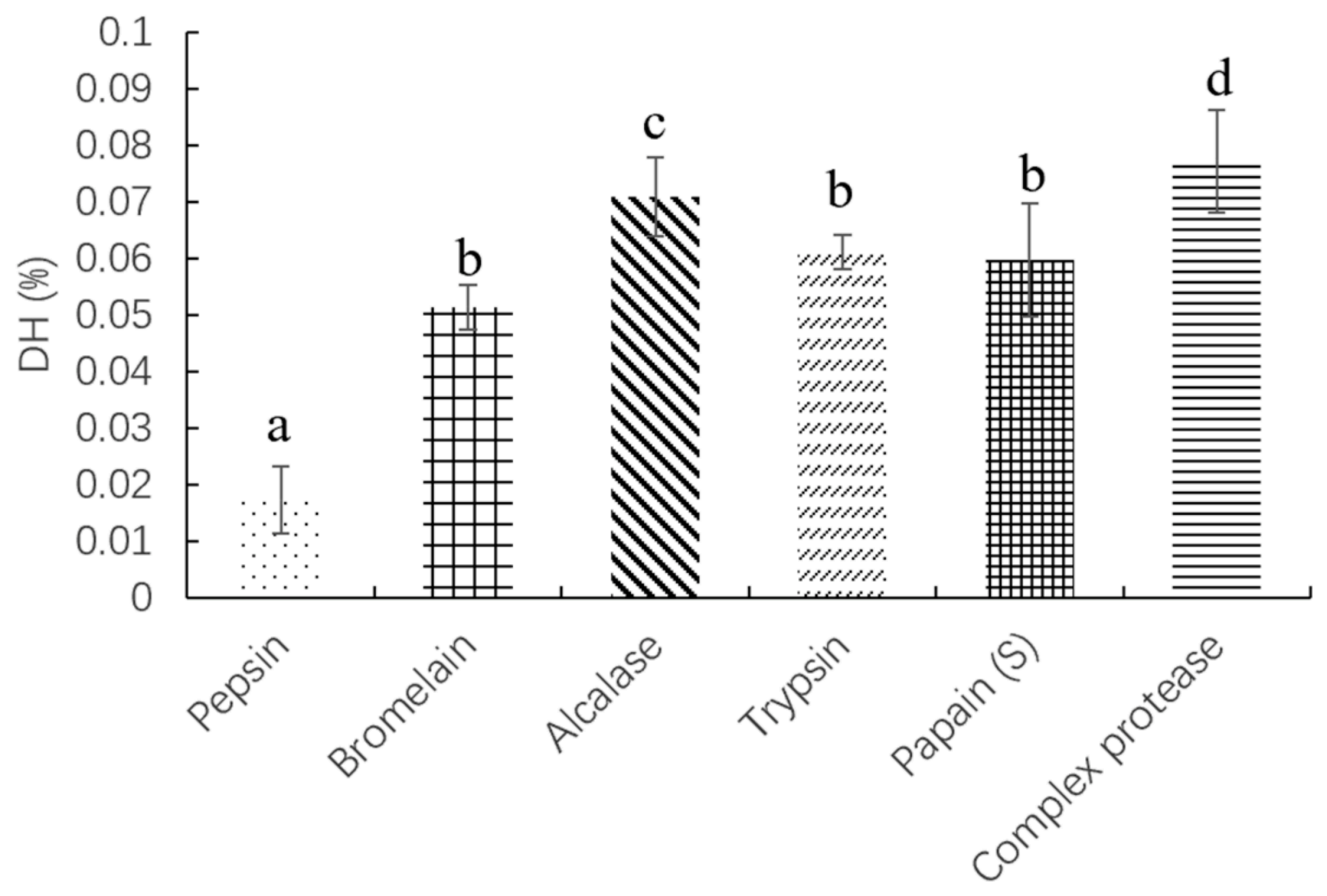
Degree of hydrolysis of goat milk protein hydrolyzed by various proteases. Different letters represent significance analysis.

According to the calculation, the degrees of hydrolysis (DH) of the hydrolysates were determined as follows: pepsin (1.73%), bromelain (5.13%), alkaline protease (7.07%), trypsin (6.11%), papain (5.96%), and protamex (7.71%), as shown in Figure 4.

### 2.4. Indirect ELISA Recognition of Cross-Allergenic Results in Cow and Goat Milk

#### 2.4.1. Recognition of Hydrolysates by Bovine α-Casein Antibody

**Figure 5:**
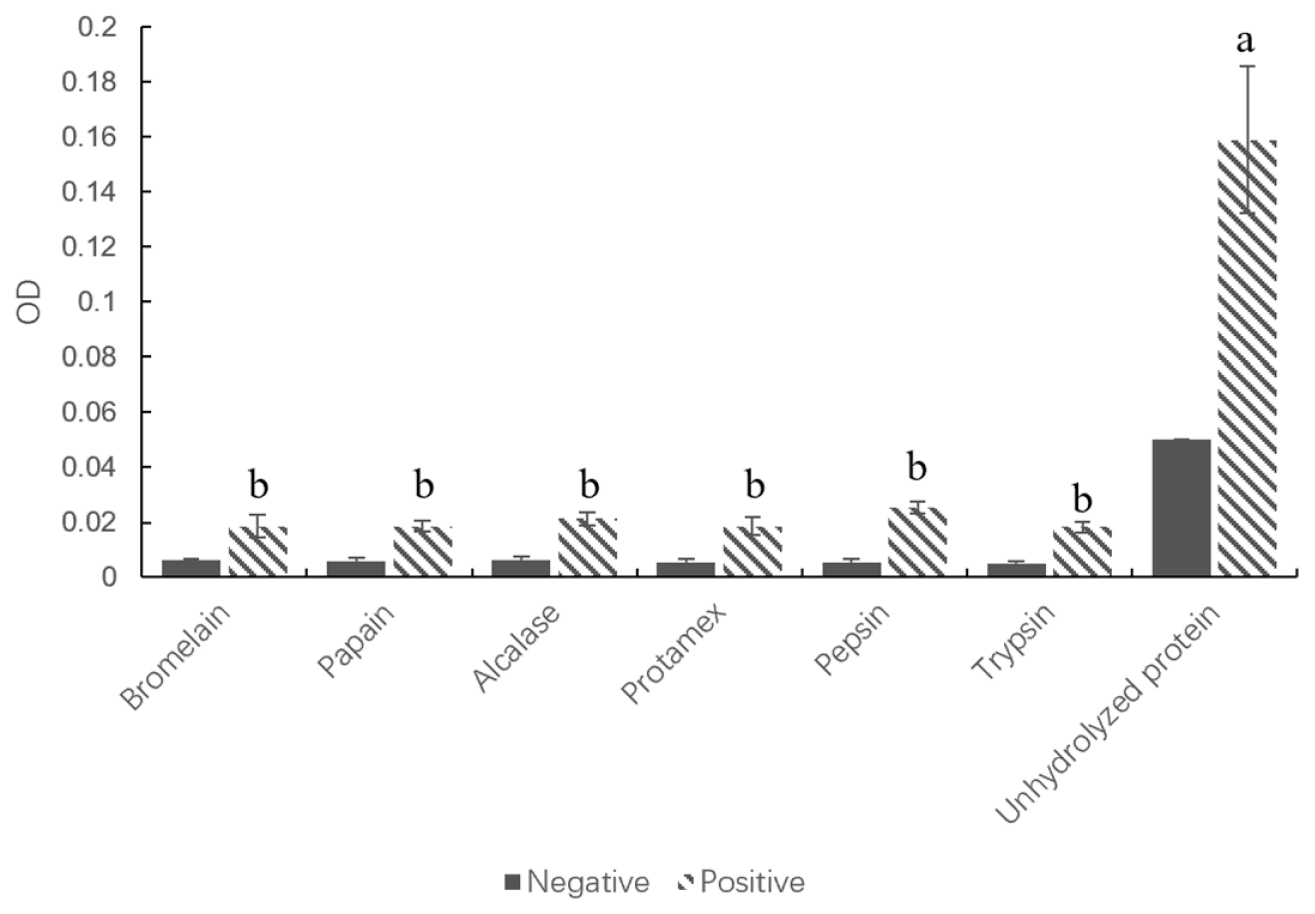
Immunoreactivity of the six enzymatic hydrolysates recognized by bovine α-casein antibody.

The residual antigen content in the six hydrolysates was assessed using a rabbit polyclonal antibody against bovine α-casein, and the results were shown in Figure 5. Compared with the unhydrolyzed protein, the OD values of positive serum for all six hydrolysates decreased significantly (*p* < 0.05). The OD values of positive serum, in descending order, were as follows: pepsin group > alkaline protease group > protamex group > papain group > bromelain group > trypsin group. These results indicate that hydrolysis with all six enzymes could, to varying extents, reduce the cross-immunoreactivity of goat milk proteins with bovine α-casein antibodies. Different letters above the bars indicate significant differences among groups (*p* < 0.05).

#### 2.4.2. Recognition of Hydrolysates by Bovine β-Casein Antibody

**Figure 6:**
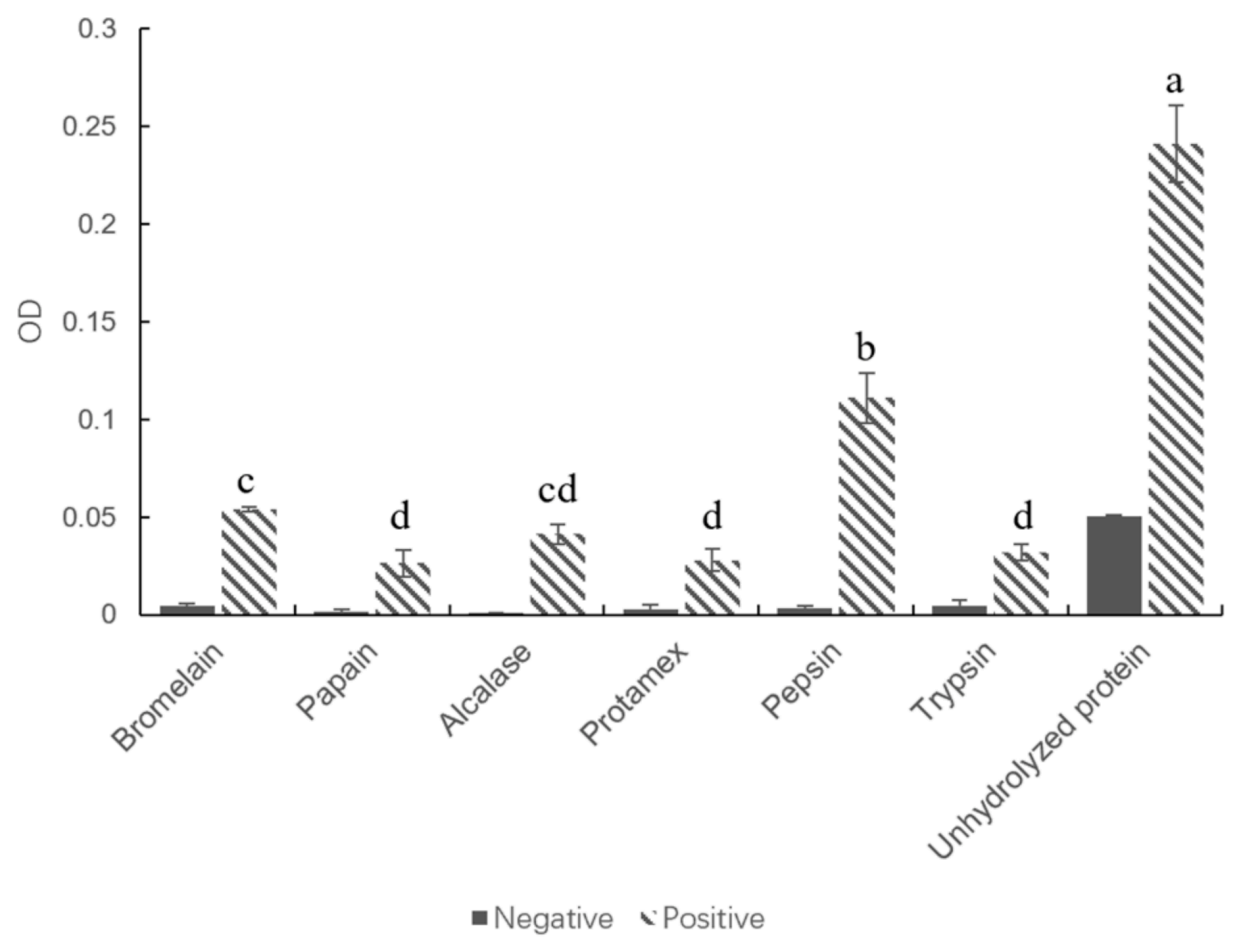
Immunoreactivity of the six enzymatic hydrolysates recognized by bovine β-casein antibody.

The residual antigen content in the six hydrolysates was assessed using rabbit polyclonal antibody against bovine β-casein, and the results were shown in Figure 6. Compared with the unhydrolyzed protein, the OD values of positive serum for all six hydrolysates decreased significantly (*p* < 0.05). The OD values of positive serum, in descending order, were as follows: pepsin group > bromelain group > alkaline protease group > trypsin group > protamex group > papain group. These results indicate that hydrolysis with all six enzymes could reduce the immunoreactivity of goat milk proteins with bovine β casein antibodies to varying degrees. Papain and protamex showed the best efficacy, bromelain, alkaline protease, and trypsin showed moderate efficacy, and pepsin showed relatively poor efficacy. Different letters above the bars indicate significant differences among groups (*p* < 0.05).

#### 2.4.3. Recognition of Hydrolysates by Bovine κ-Casein Antibody

**Figure 7:**
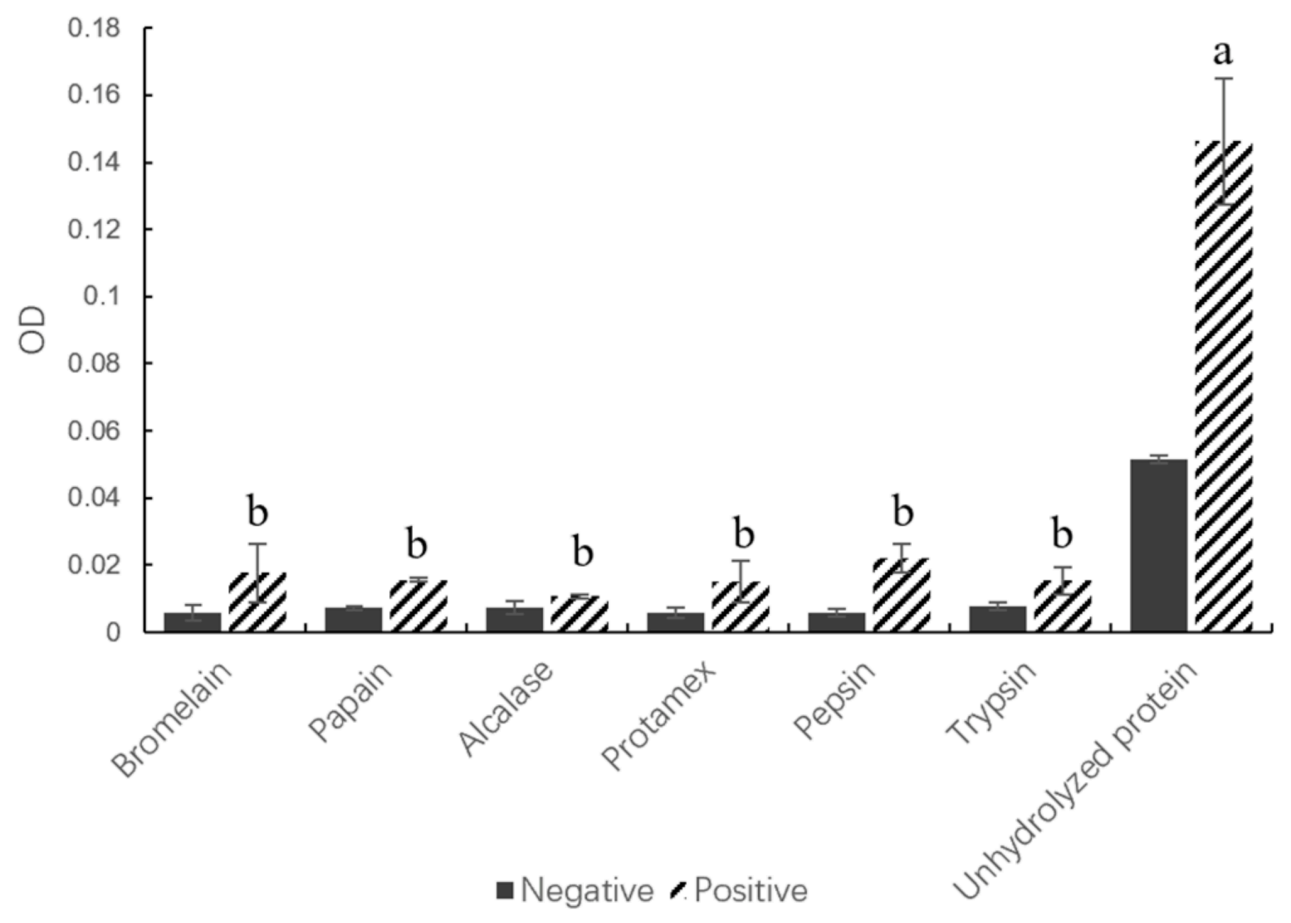
Immunoreactivity of the six enzymatic hydrolysates recognized by bovine κ-casein antibody.

The residual antigen contents in the six hydrolysates were identified by ELISA using a rabbit polyclonal antibody against bovine κ-casein. Duncan’s test was used for significance analysis, and different letters indicated significant differences among groups. The negative serum and positive serum for the unhydrolyzed protein showed significant differences (*p* < 0.05), indicating that the unhydrolyzed protein possessed strong immunoreactivity. The residual antigen content in the six hydrolysates was assessed using rabbit polyclonal antibody against bovine κ-casein, and the results are shown in Figure 7. Compared with the unhydrolyzed protein, the OD values of positive serum for all six hydrolysates decreased significantly (*p* < 0.05). The OD values of positive serum, in descending order, were as follows: pepsin group > bromelain group > papain group > trypsin group > protamex group > alkaline protease group. These results indicate that hydrolysis with all six enzymes could reduce the immunoreactivity of goat milk proteins with bovine κ-casein antibodies to varying degrees. Different letters above the bars indicate significant differences among groups (*p* < 0.05). 

#### 2.4.4. Recognition of Hydrolysates by Bovine α-Lactalbumin Antibody

**Figure 8:**
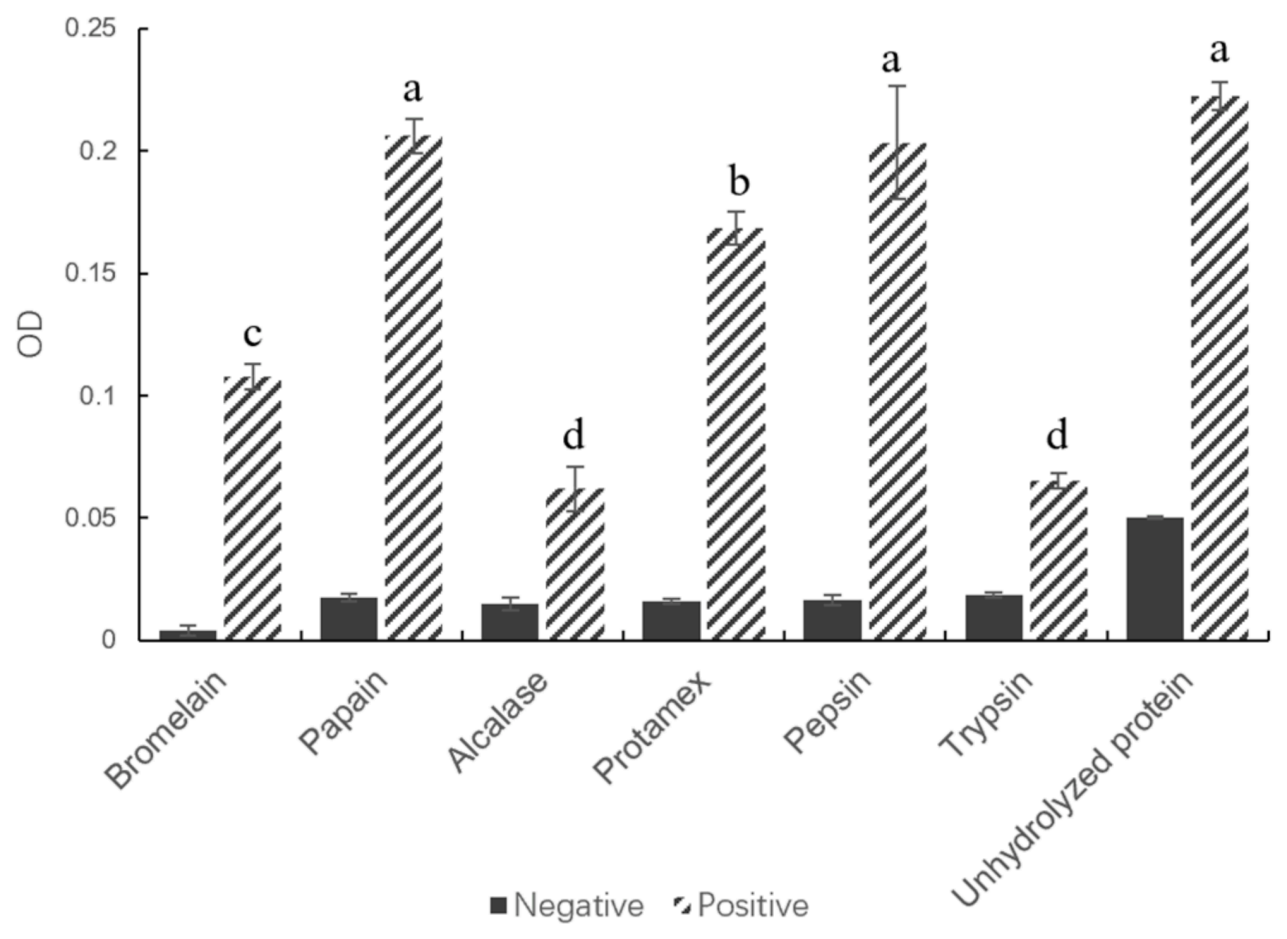
Immunoreactivity of the six enzymatic hydrolysates recognized by bovine α-lactalbumin antibody.

The residual antigen content in the six hydrolysates was assessed using rabbit polyclonal antibody against bovine α-lactalbumin, and the results are shown in Figure 8. Compared with the unhydrolyzed protein, the OD values of positive control serum for all hydrolysates except papain and pepsin decreased significantly (*p* < 0.05). The OD values of positive serum, in descending order, were as follows: protamex group > bromelain group > trypsin group > alkaline protease group. These results indicate that hydrolysis with these four enzymes could reduce the immunoreactivity of goat milk proteins with bovine α-lactalbumin antibodies to varying degrees. Among them, alkaline protease and trypsin showed the best efficacy, bromelain showed moderate efficacy, and protamex showed relatively poor efficacy. Different letters above the bars indicate significant differences among groups (*p* < 0.05).

#### 2.4.5. Recognition of Hydrolysates by Bovine β-Lactoglobulin Antibody

**Figure 9:**
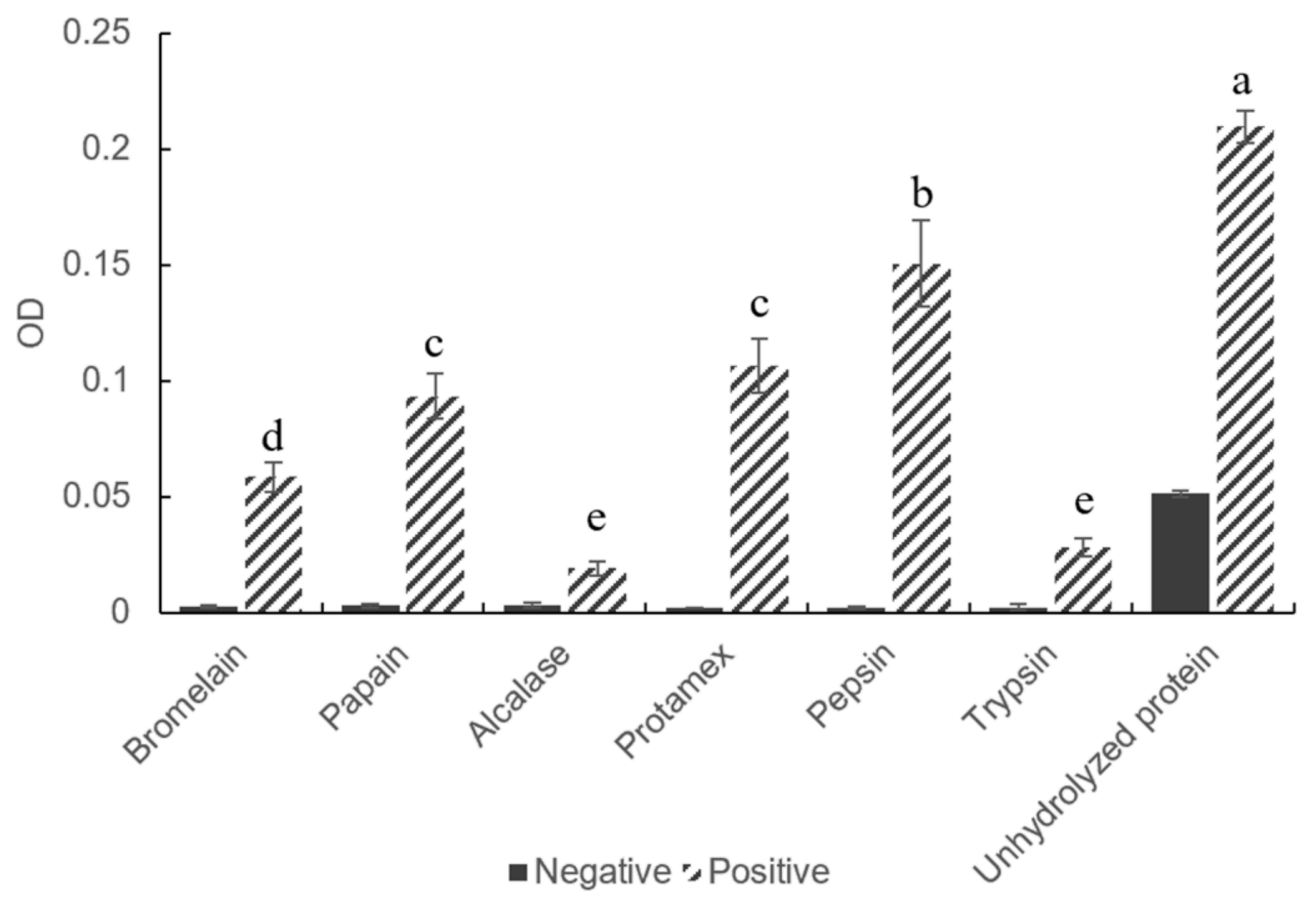
Immunoreactivity of six enzymatic hydrolysates recognized by β-lactoglobulin antibody.

The residual antigen content in the six hydrolysates was assessed using rabbit polyclonal antibody against bovine β-lactoglobulin, and the results are shown in Figure 9. Compared with the unhydrolyzed protein, the OD values of positive serum for all six hydrolysates decreased significantly (*p* < 0.05). The OD values of positive serum, in descending order, were as follows: pepsin group > protamex group > papain group > bromelain group > trypsin group > alkaline protease group. These results indicate that hydrolysis with all six enzymes could reduce the immunoreactivity of goat milk proteins with bovine β-lactoglobulin antibodies to varying degrees. Among them, alkaline protease and trypsin showed the best efficacy, bromelain showed moderate efficacy, papain and protamex showed moderate-to-poor efficacy, and pepsin showed relatively poor efficacy. Different letters above the bars indicate significant differences among groups (*p* < 0.05).

### 2.5. Mass Spectrometry Analysis of Amino Acid Sequences of Peptides in Hydrolysates

#### 2.5.1. Alignment Results of Peptide Amino Acid Sequences Identified by Mass Spectrometry

The mass spectrometry results (Table 5) showed that the peptides detected in the six protease hydrolysates overlapped to varying extents with the B-cell linear epitope regions predicted in Section 2, confirming that the enzymatic cleavage sites were located within these predicted cross-reactive regions. When combined with the reduced antibody binding observed in the ELISA assays (Figure 5, Figure 6, Figure 7, Figure 8 and Figure 9), these findings support that hydrolysis within these epitope regions contributed to the decreased immunoreactivity. The hydrolysates from different proteases exhibited marked differences in epitope coverage and peptide numbers, reflecting the distinct cleavage specificities of each enzyme.

In the bromelain hydrolysate, two overlapping peptides were identified. FAWPQY was located within the 173–192 region of α_S2_-casein, which was identified as a cross-reactive epitope in the similar-sequence prediction between cow and goat milk; LKDLKDY fell within the predicted epitope region of 11–30 of α-lactalbumin.

The protamex hydrolysate yielded only one overlapping peptide, LAMAAS, corresponding to the 36–55 epitope region of β-lactoglobulin. Although this enzyme exhibited the highest DH among the six proteases, its epitope coverage was the narrowest, suggesting that its hydrolytic action was rather dispersed and did not preferentially target critical antigenic regions.

The alkaline protease hydrolysate yielded three overlapping peptides. PPQSVLS was located in the 314–332 region of β-casein, and IPIQYVLS and LPYPYY both fell within the predicted epitopes of κ-casein (39–58 and 74–93, respectively). Although the number of overlapping peptides identified was not large, each was situated in the core regions of the cross-reactive epitopes predicted earlier, indicating that this enzyme exhibited high epitope targeting specificity.

The trypsin hydrolysate yielded two overlapping peptides. YLGYLEQL corresponded to the 101–119 epitope of α_S1_-casein, and AMAASDISLL covered the 36–55 region of β-lactoglobulin. Both are highly conserved sequence regions among the three milk sources, and disruption of these sites by enzymatic hydrolysis effectively reduces cross-reactivity.

The papain hydrolysate yielded three overlapping peptides, covering three different allergens. NEINQFYQK was located in the 94–113 region of α_S2_-casein; FQSEEQQQTEDELQDK corresponded to the 193–212 epitope of β-casein; and AMAASDISL fell within the 36–55 interval of β-lactoglobulin. This enzyme yielded epitope-spanning peptides from three different proteins, demonstrating relatively broad cross-reactive epitope coverage.

In the pepsin hydrolysate, no peptide matching the predicted epitopes was detected by mass spectrometry analysis. Combined with its overall poor hypoallergenic efficacy in the ELISA experiments, this further indicates that pepsin has limited efficiency in cleaving the linear cross-reactive epitopes between cow and goat milk.

Overall, the hydrolysates from alkaline protease, trypsin, and papain all contained peptides located within the predicted cross-reactive epitopes, providing molecular-level evidence that these regions were subjected to enzymatic cleavage. The concomitant reduction in antibody binding (ELISA) further indicated that such cleavage contributed to the loss of epitope functionality.

#### 2.5.2. Mass Spectra

The mass spectra of selected peptide fragments from the bromelain, complex protease, alkaline protease, trypsin, and papain hydrolysates are shown in Figure 10, Figure 11, Figure 12, Figure 13, Figure 14, Figure 15, Figure 16, Figure 17, Figure 18, Figure 19 and Figure 20, respectively.

Bromelain-derived peptides: FAWPQY (177–182) and LKDLKDY (12–18).

Protamex-derived peptides: LAMAAS (22–27).

Alcalase-derived peptides: IPIQYVLS (26–33), PPQSVLS (177–183), LPYPYY (84–89).

Trypsin-derived peptides: YLGYLEQL (91–98), AMAASDISLL (23–32).

Papain (s)-derived peptides: NEINQFYQK (84–92), FQSEEQQQTEDELQDK (33–48), AMAASDISL (21–31).

## 3. Discussion

Research on cross-reactivity between goat and cow milk allergens has progressed continuously, evolving from phenomenological observations to mechanistic explorations, from whole proteins to specific epitopes, and from single-species to multi-species comparisons. Bellioni-Businco et al. [23] reported that bovine and caprine α-caseins share 87–98% sequence identity. In the present study, we found that the amino acid sequence similarities of the six homologous allergen proteins (α_S1_-casein, α_S2_-casein, β-casein, κ-casein, α-lactalbumin, and β-lactoglobulin) between cow milk and goat milk and between cow milk and sheep milk all exceeded 85%, and five to nine conserved homologous sequence regions were identified in each allergen. It should be noted, however, that sequence similarity does not necessarily lead to cross-reactivity. Hazebrouck et al. [24] found that some patients were allergic only to caprine β-casein while tolerating bovine β-casein, despite 91% sequence homology, indicating that subtle differences in antigenic epitopes can be sufficient to alert antibody recognition. In this study, through cross-validation between SVMTriP epitope prediction and BLASTP alignment, we further screened epitope regions within these similar sequences that are likely to be directly involved in antibody binding. For instance, although the overall similarity of α_S2_-casein between cow milk and goat milk exceeded 90%, among the eight similar segments identified here, the AA97–107 and AA180–190 regions were not fully conserved between the two milk sources; such minor differences may serve as the structural basis for differential immune recognition, consistent with the conclusion of Restani et al. [25] that phylogenetic relatedness influences the intensity of cross-reactivity.

Järvinen et al. [26] defined IgE epitopes of all six major cow’s milk allergens and identified key regions associated with persistent CMA (e.g., αs1-casein AA123–132, αs2-casein AA171–180, κ-casein AA155–164); Lisson et al. [27], who mapped casein epitopes across cow, goat, and buffalo; Maynard et al. [28], who identified α-lactalbumin 17–58 as a major IgE-binding region; and Hu et al. [29], who confirmed β-lactoglobulin AA30–43 as an IgE linear epitope. Our predicted regions showed good overlap with these validated epitopes. For example, our αs1-casein AA101–119 (containing YLGYLEQL) overlaps with the AA123–132 region; our β-lactoglobulin AA36–55 (containing AMAASDISLL) covers the AA30–43 epitope; and our αs2-casein AA173–192 (containing FAWPQY) matches the AA171–180 region reported by Järvinen et al. These overlaps support the biological relevance of our targets.

The hydrolysis efficiencies of different proteases varied significantly, and these differences were determined by both the cleavage specificities of the enzymes and the structures of the allergenic proteins. The SDS-PAGE results showed that the hydrolysates obtained with alkaline protease and trypsin exhibited almost no visible protein bands, indicating that the large-molecular-weight proteins were thoroughly degraded into small peptides; in contrast, the hydrolysates from bromelain and pepsin still displayed numerous protein bands, reflecting a relatively limited hydrolysis extent, which was generally consistent with the degree-of-hydrolysis measurements. The distinct action characteristics of the enzymes explained these observations. Alkaline protease preferentially cleaved at the carboxyl side of hydrophobic amino acids (S, G, Y, F, W), and thus exhibits high hydrolysis efficiency towards caseins, which are highly hydrophobic proteins. Trypsin specifically cleaved at the carboxyl side of lysine and arginine residues, which are widely distributed in milk proteins. Although pepsin showed a low degree of hydrolysis, it cleaved specifically at hydrophobic amino acids (Phe, Leu) under acidic conditions and could still break down large proteins into small peptides. The nearly complete disappearance of electrophoretic bands for pepsin, despite its low DH, may be related to the uniform molecular-weight distribution of its hydrolysates, which are mainly small peptides. The degree of hydrolysis reflects only the content of free amino groups and does not fully characterize the molecular-weight distribution of the products.

In the hydrolysis experiment section, we acknowledge that we did not perform a dedicated control experiment (incubating milk proteins without enzyme at pH 2.0, 50 °C, and 60 °C) to confirm the absence of non-enzymatic acid/thermal hydrolysis. While a dedicated no-enzyme control was not performed, the available data (especially the pepsin result) indicate that non-enzymatic hydrolysis was minimal. If acid hydrolysis were substantial, we would expect the pepsin hydrolysate to show considerable protein breakdown. In fact, it exhibited the lowest DH (1.73%) among all enzymes and retained numerous visible protein bands on SDS-PAGE (Figure 2, lane 5), indicating that acid alone did not cause significant hydrolysis. Both bromelain (50 °C) and alkaline protease (60 °C) treatments resulted in extensive hydrolysis, while pepsin (37 °C) gave minimal hydrolysis, showing that hydrolysis was driven by enzyme specificity rather than temperature alone. The DH values correlated well with the SDS-PAGE patterns and with each enzyme’s known cleavage specificity, further supporting that the observed effects were due to enzymatic activity rather than non-enzymatic degradation. Future studies should include such controls to further validate the interpretation.

Protamex exhibited the highest degree of hydrolysis (7.71%), yet its efficacy on reducing immunoreactivity in the ELISA experiments was not optimal, and only one overlapping peptide (LAMAAS) located in the β-lactoglobulin epitope region was identified by mass spectrometry, giving the narrowest epitope coverage. This is because the DH reflects the overall extent of peptide-bond cleavage, but only cleavage occurring within antigenic epitope regions can effectively reduce immunoreactivity. Protamex was a non-specific endopeptidase, and its hydrolytic action may have been uniformly distributed over the protein surface and interior rather than being concentrated on immunodominant epitopes. Therefore, although a large number of small peptides were generated, key antibody-recognition regions may have remained intact. The molecular weight of peptides obtained after hydrolysis also exerts differential effects on antigenicity, although the optimal molecular weight varies depending on the hydrolysis process employed, the ultimate impact on antigenicity may be modulated by enzyme specificity, patient sensitivity to antigens, and the optimization of hydrolysis conditions. Refs. [30,31], suggesting that future enzymatic process design should focus more on epitope coverage rather than merely pursuing a high DH.

In contrast, although the mass spectrometry of the alkaline protease hydrolysate identified only three overlapping peptides, all of them fell within the core regions of the predicted cross-reactive epitopes (PPQSVLS in the β-casein epitope, and IPIQYVLS and LPYPYY in the κ-casein epitopes), and alkaline protease exhibited consistent efficacy in the ELISA assays for all five allergens. This indicates that alkaline protease exhibited favorable epitope targeting specificity—its preferred cleavage sites (at the carboxyl side of hydrophobic amino acids such as S, G, Y, F, and W) were precisely enriched within the cross-reactive epitope regions. This finding was consistent with the report by Hahn et al. [32] that alkaline protease effectively reduced the immunoreactivity of goat milk proteins. Trypsin exhibited excellently towards α-lactalbumin and β-lactoglobulin, and the mass spectrometry-identified peptides YLGYLEQL (α_S1_-casein epitope) and AMAASDISLL (β-lactoglobulin epitope) were both located in highly conserved regions. Trypsin specifically cleaved at the carboxyl side of lysine and arginine residues, and these two basic amino acids were densely distributed within the epitope regions of α-lactalbumin and β-lactoglobulin, explaining its selective advantage for particular allergens. Papain was the most effective in reducing cross-reactivity towards β-casein; the mass spectrometry-identified peptide FQSEEQQQTEDELQDK was located in the core epitope region (AA193–212) of β-casein, which was highly conserved among the three milk sources. Papain’s preference for L, G, K, and R residues may have precisely cleaved this critical region. However, papain showed limited efficacy against α-lactalbumin, suggesting that the distribution of its cleavage sites within the α-lactalbumin epitope region was insufficient to effectively disrupt the antibody-recognition conformation. Pepsin showed the poorest performance across all evaluation criteria, and no epitope-matching peptides were detected by mass spectrometry. This may be related to the substrate conformational changes induced by its acidic working environment (pH 2.0) [33]. Although pepsin can efficiently cleave at hydrophobic amino acid sites under strongly acidic conditions, the excessively acidic environment may cause irreversible conformational alterations in the proteins, thereby “burying” certain linear epitopes or promoting the formation of aggregated structures resistant to enzymatic cleavage. This finding suggests that when evaluating enzymatic hydrolysis effects, the influence of the hydrolysis conditions themselves on substrate conformation should be considered, rather than focusing solely on enzyme specificity.

In summary, the differences in the ability of reducing immunoreactivity among the various proteases essentially reflect the degree of matching between the enzymes’ cleavage specificity and the amino acid composition of the allergen epitopes. Enzymatic processes for low-allergenicity products should be designed to employ proteases whose cleavage sites match the sequence characteristics of the target allergens, rather than simply pursuing a high DH.

The distribution of overlapping peptides identified by mass spectrometry showed a clear protease-dependent pattern. The three overlapping peptides from alkaline protease were located within the core regions of the predicted epitopes of β-casein (PPQSVLS) and κ-casein (IPIQYVLS and LPYPYY), which was fully consistent with the excellent desensitization efficacy of alkaline protease towards β-casein and κ-casein in the ELISA assays. Notably, IPIQYVLS and LPYPYY were situated in two separate epitope regions of κ-casein (AA39–58 and AA74–93, respectively), indicating that alkaline protease achieved effective cleavage at multiple immunodominant sites of κ-casein, thereby thoroughly disrupting the antibody-recognition capacity of this protein. The two overlapping peptides from trypsin (YLGYLEQL and AMAASDISLL) were both located in highly conserved regions of α_S1_-casein and β-lactoglobulin. The α_S1_-casein AA101–119 region containing YLGYLEQL was completely identical across cow, goat, and sheep milk, representing a core cross-reactive epitope; the β-lactoglobulin AA36–55 region containing AMAASDISLL was also highly conserved. The precise cleavage of these two critical regions by trypsin provided a molecular explanation for its strong performance against α-lactalbumin and β-lactoglobulin in the ELISA assays and for the lowest specific antibody levels observed in the animal experiments. The three overlapping peptides from papain covered three different allergens—α_S2_-casein (NEINQFYQK), β-casein (FQSEEQQQTEDELQDK), and β-lactoglobulin (AMAASDISL)—giving papain the broadest epitope coverage among the six enzymes. This feature was consistent with its outstanding performance towards β-casein in the ELISA assays and also explained why the papain hydrolysate exhibited the lowest clinical symptom scores and histamine release in animal experiments: extensive destruction of multiple allergen epitopes may have reduced overall the capacity to form sensitizing complexes. The two overlapping peptides from bromelain (FAWPQY in the α_S2_-casein epitope and LKDLKDY in the α-lactalbumin epitope), though limited in number, were both located within the predicted epitope regions, indicating that although its DH was low (5.13%) and its cleavage of the limited epitopes was precise. Pepsin did not yield any matching peptide, which was consistent with its poorest performance across all evaluation criteria and further confirmed its extremely limited efficiency in cleaving cross-reactive linear epitopes.

The mass spectrometry results also revealed an important phenomenon: the overlapping peptides identified in this study were generally short (6–16 amino acids), which was consistent with the residual fragments generated after limited cleavage within the epitope regions by each protease. These short peptides, having lost the complete spatial conformation and critical contact residues required for antibody binding, could not be effectively recognized by IgE antibodies, representing the molecular essence of the reduction in immunoreactivity by enzymatic hydrolysis. Li et al. [33] observed similar phenomena when analyzing goat whey protein hydrolysates by LC-MS/MS and noted that peptides shorter than 20 amino acids generally lose full epitope functionality.

It is important to note that the MS detection of epitope-overlapping peptides per se confirms peptide bond hydrolysis within the epitope regions, but does not independently prove the complete loss of epitope function. The functional inactivation of these epitopes is primarily supported by the concurrent significant reduction in antibody binding in the ELISA experiments. The combination of MS (structural cleavage) and ELISA (functional loss) provides compelling evidence that enzymatic hydrolysis effectively disrupts the cross-reactive epitopes. As previously reported, peptides shorter than 20 amino acids often lack sufficient contact residues for stable antibody recognition, further corroborating that the observed cleavage likely abrogated epitope activity.

We acknowledge that this study has several limitations. First, the polyclonal antibodies used in this study were derived from rabbit immune sera, and rabbit Immunoglobulin G (IgG) differs from human IgE in epitope-recognition patterns. Although rabbit polyclonal antibodies can effectively reflect overall changes in protein antigenicity, human IgE-recognized epitopes are subject to individual variation. Our ELISA results, obtained with rabbit polyclonal antibodies, indicate a reduction in antigenic reactivity rather than a direct confirmation of reduced allergenicity in humans. Therefore, we need further validation using sera from cow’s milk-allergic individuals would be necessary to confirm the hypoallergenic potential of the hydrolysates. Future studies should validate the findings using sera from patients with cow’s milk allergy, in order to more accurately assess the clinical desensitization efficacy of the enzymatic hydrolysates. Second, this study employed only a single-enzyme hydrolysis strategy and did not explore the synergistic effects of combined or sequential enzymatic treatments. Alkaline protease, trypsin, and papain each exhibited outstanding performance against different allergens, and theoretically, combining two or three enzymes in a specific sequence could achieve complementary effects. Li et al. [34] have preliminarily confirmed that multi-enzyme synergistic hydrolysis can improve the peptide molecular-weight distribution while maintaining low allergenicity. Future studies could further optimize the sequence and ratio of enzymatic treatments based on these findings and systematically evaluate the immunological properties of the multi-enzyme hydrolysates. It should also be noted that the proteases themselves—papain, alcalase, pepsin, trypsin, and bromelain—are proteins and may possess inherent allergenic potential. Although heat inactivation (85 °C, 10 min) and the extensive hydrolysis process are expected to substantially degrade the enzyme molecules, as indicated by the absence of visible protease bands on SDS-PAGE (Figure 2), we did not directly assess the residual antigenicity of the proteases. Future studies should evaluate this aspect, particularly if the hydrolysates are intended for use in hypoallergenic infant formulas or other food products for sensitive populations.

## 4. Materials and Methods

### 4.1. Materials and Reagents

L-Leucine, o-phthalaldehyde, papain, bromelain, Bovine serum albumin (BSA) and the Gly-Gly-Tyr-Arg standard were purchased from Shanghai Yuanye Biotechnology Co., Ltd. (Shanghai, China). N-Acetylcysteine was obtained from Biotopped (Beijing, China). Alcalase, protamex, and trypsin were purchased from Novozymes (Copenhagen, Denmark), while pepsin was obtained from Amano Enzyme (Nagoya, Japan). Glycine, sodium dodecyl sulfate (SDS), Coomassie Brilliant Blue R-250, glacial acetic acid, Tween-20, and methanol were purchased from Beijing Banxia Science & Technology Development Co., Ltd. (Beijing, China). 4-Chloro-1-naphthol, dimethyl sulfoxide, acrylamide, N,N′-methylenebisacrylamide, β-mercaptoethanol, amido black, glycerol, and 3,3′,5,5′-tetramethylbenzidine (TMB) were obtained from Sigma-Aldrich (St. Louis, MO, USA). Phosphoric acid was purchased from Tianjin Chemical Reagent Co., Ltd. (Tianjin, China). Concentrated sulfuric acid and concentrated hydrochloric acid were obtained from Modern Oriental Science and Technology Development Co., Ltd. (Beijing, China). Sodium tartrate, copper sulfate, ammonium carbonate, and magnesium chloride hexahydrate were purchased from Tianjin Fuchen Chemical Reagent Co., Ltd. (Tianjin, China). The Horseradish Peroxidase (HRP)-conjugated goat anti-rabbit IgG secondary antibody was purchased from Beijing Youyizhonglian Biotechnology Co., Ltd. (Beijing, China). The low-molecular-weight protein standard was obtained from TIANGEN Biotech (Beijing, China). Anhydrous ether and sodium bicarbonate were obtained from Beijing Merida Technology Co., Ltd. (Beijing, China).

### 4.2. Instruments and Equipment

The following instruments were used in this study: a UV-Vis spectrophotometer (Hitachi, Tokyo, Japan), a PHS-3C pH meter (Shanghai Yidian Scientific Instrument Co., Ltd., Shanghai, China), a DYY-7C electrophoresis apparatus (Beijing Liuyi Instrument Factory, Beijing, China), a SHZ-C water bath constant temperature shaker (Shanghai Longyue Instrument Equipment Co., Ltd., Shanghai, China), an SH-2 magnetic stirrer (Beijing Dongfang Kaiwu Scientific Apparatus Co., Ltd., Beijing, China), a BSA124S-CW electronic balance (Sartorius Scientific Instruments Co., Ltd., Beijing, China), a Q Exactive Plus liquid chromatography-mass spectrometry system (Thermo Fisher Scientific, Waltham, MA, USA), an electrophoresis gel documentation and analysis system (Bio-Rad, Hercules, CA, USA), a freeze dryer (Labconco, Kansas City, MO, USA), a low-temperature high-speed centrifuge (Sigma, Springfield, MO, USA), a KHB ST-360 microplate reader (Shanghai Kehua Bio-engineering Co., Ltd., Shanghai, China), a rotary evaporator RE-2000A (Shanghai Yarong Biochemical Instrument Factory, Shanghai, China), and a constant temperature drying oven (Shanghai Yiheng Scientific Instrument Co., Ltd., Shanghai, China).

### 4.3. Methods

#### 4.3.1. BLAST Analysis of Cross-Allergen Sequence Similarity Between Cow, Goat, and Sheep Milk

The complete amino acid sequences of the major allergens (α_S1_-casein, α_S2_-casein, β-casein, κ-casein, α-lactalbumin, and β-lactoglobulin) from the three milk sources were retrieved from the Allergome allergen database (http://www.allergome.org/, accessed on 23 December 2024) [35] and the NCBI public database (http://www.ncbi.nlm.nih.gov/, accessed on 23 December 2024) [36]. B-cell linear epitopes of these allergenic proteins were then predicted using the SVMTriP online prediction platform (http://sysbio.unl.edu/SVMTriP/, accessed on 23 December 2024) [21], and peptide regions with confidence scores above the threshold were retained. Finally, pairwise sequence alignments were performed using the BLASTP algorithm on the NCBI platform (http://blast.ncbi.nlm.nih.gov/Blast.cgi, accessed on 23 December 2024) [37] to evaluate the possibility of cross-reactivity according to the Codex Alimentarius Commission criteria, and to identify conserved regions among allergens from different milk sources.

#### 4.3.2. Preparation of Whole Goat Milk Protein Powder and Its Hydrolysates

Goat milk was centrifuged at 4 °C and 8000 rpm for 20 min to remove most of the milk fat. The defatted goat milk was then preheated in a water bath at 50–55 °C and centrifuged again at 6000–15,000 rpm to reduce the fat content to below 0.06% (*w*/*w*). The twice-defatted goat milk was freeze-dried to obtain whole goat milk protein powder. An appropriate amount of whole goat milk protein powder was weighed and dissolved to prepare a 3% (*w*/*v*) protein solution. The reaction conditions for the thermostatic water-bath shaker were set according to the optimal temperature and pH parameters provided by the protease suppliers (Table 6). Subsequently, six proteases with different cleavage specificities—namely, protamex, alkaline protease, pepsin, trypsin, papain, and bromelain—were added at an enzyme-to-substrate ratio of 1000 U/g protein to hydrolyze the whole goat milk protein under their respective optimal conditions for 2 h [38].

#### 4.3.3. SDS-PAGE

A 12.5% separating gel and a 4.5% stacking gel were prepared. Samples were mixed with loading buffer at a 1:1 ratio and boiled in a water bath for approximately 10 min. Each well was loaded with 20 μL of sample. Electrophoresis was carried out at a constant current of 20 mA, and the duration was controlled by monitoring the position of the tracking dye. After electrophoresis, the gel was stained with Coomassie Brilliant Blue R-250 for 30 min, followed by destaining until the protein bands were clearly visible.

#### 4.3.4. Determination of the Degree of Hydrolysis

A 3.2 mL aliquot of freshly prepared OPA reagent was accurately transferred into a test tube, and 400 μL of the hydrolysate or L-leucine standard solution was added. The absorbance was measured at 340 nm using a UV-Vis spectrophotometer [39]. A standard curve was constructed using L-leucine as the standard. The total free amino group content of the samples after complete acid hydrolysis was determined using an amino acid analyzer according to the Chinese national food safety standard GB 5009.124-2016 [40], “Determination of Amino Acids in Foods”. The degree of hydrolysis (DH) was calculated using the following formula:

$$\mathrm{DH}\;=\;\frac{({\mathrm{NH}}_{2})T_{X}-({\mathrm{NH}}_{2})T_{0}}{({\mathrm{NH}}_{2})\mathrm{Total}-({\mathrm{NH}}_{2})T_{0}}$$

where (NH_2_)T_x_ is the free amino group content of the enzymatic hydrolysate (μmol/mL), (NH_2_)T_0_ is the free amino group content of the unhydrolyzed goat milk protein (μmol/mL), and (NH_2_)Total is the total free amino group content of the goat milk protein sample (μmol/mL).

#### 4.3.5. Assessment of Cross-Reactivity Between Cow and Goat Milk Proteins

The synthesized similar sequences were assayed in a 96-well microplate; each well was first coated with streptavidin, then sequentially incubated with the sequences for epitope mapping, and finally underwent antibody detection [41]. The diluted antigen coating solution was added to the microplate wells and incubated overnight at 4 °C. After washing with Phosphate Buffered Saline with Tween-20 (PBST), blocking buffer (1% BSA in PBST) was added and the plate was incubated at 37 °C for 1 h. The primary antibody was then added and incubated at 37 °C for 2 h, followed by washing. The secondary antibody (goat anti-rabbit IgG) was added to the wells and incubated at 37 °C for 1 h, after which the plate was washed again. Color development was performed using substrate solution, and the reaction was terminated with 2 mol/L sulfuric acid. The absorbance of each well was measured at 450 nm (OD_450_).

The residual antigen contents in the six hydrolysates were identified by ELISA using a rabbit polyclonal antibody against six bovine milk protein. Duncan’s test was used for significance analysis, and different letters indicated significant differences among groups. The negative serum and positive serum for the unhydrolyzed protein showed significant differences (*p* < 0.05), indicating that the unhydrolyzed protein possessed strong immunoreactivity.

#### 4.3.6. Mass Spectrometry Analysis of Hydrolysates

Each sample (1 mg) was accurately weighed and dissolved in an appropriate solvent to obtain a protein solution at 1 mg/mL. This solution was filtered through a 10 kDa ultrafiltration membrane and then vacuum-freeze-dried. The dried samples were redissolved in 0.1% formic acid in water, and the concentration of each sample was adjusted to 500 ng/μL; the injection volume was set to 2 μL.

Peptide separation was performed using a Vanquish Neo nano-liquid chromatography system (Thermo Fisher Scientific, Waltham, MA, USA). The analytical column was an Acclaim PepMap RSLC (75 μm × 25 cm), and the trap column was an Acclaim PepMap 100 (75 μm × 2 cm). Mobile phase A was ultrapure water, and mobile phase B was 80% acetonitrile in water containing 0.1% formic acid. The flow rate was set at 0.3 μL/min, and the sample load was 1000 ng (i.e., 2 μL).

Mass spectrometry analysis was carried out using a QE Plus high-resolution mass spectrometer (Thermo Fisher Scientific, Waltham, MA, USA). Data-dependent acquisition was performed in Full-MS/dd-MS^2^ mode, with a mass scan range of 250–1800 *m*/*z*. The resolution for MS1 was set to 70,000, and the automatic gain control (AGC) target was 3 × 10^6^. In each scan cycle, the top 20 most abundant precursor ions were selected for fragmentation; the resolution for MS2 was set to 17,500, and the AGC target was 1 × 10^5^. The collision energy was set to 28%, and dynamic exclusion was enabled with an exclusion time of 25 s.

The raw mass spectrometry data were processed using Proteome Discoverer 2.5 software. The search was performed against the UniProt protein database [42]. The enzyme parameters were set according to the protease used for each hydrolyzed sample. Specific cleavage sites are listed in Table 7.

## 5. Conclusions

In this study, bioinformatics prediction revealed that α_S1_-casein, α_S2_-casein, β-casein, κ-casein, α-lactalbumin, and β-lactoglobulin each contained five to nine homologous sequence segments, and these conserved regions represented the molecular basis for cross-reactivity. Indirect ELISA results showed that all six hydrolysates exhibited significantly reduced immunoreactivity against the five major cow’s milk allergens. Specifically, alkaline protease exhibited consistently strong efficacy against all five allergens; papain was most effective against β-casein; and trypsin showed pronounced efficacy against α-lactalbumin and β-lactoglobulin, where pepsin showed relatively poor efficacy. Mass spectrometry analysis further elucidated the peptide composition and epitope coverage of the different hydrolysates. Bromelain yielded two overlapping peptides (FAWPQY and LKDLKDY), protamex yielded one (LAMAAS), alkaline protease yielded three (PPQSVLS, IPIQYVLS, and LPYPYY), trypsin yielded two (YLGYLEQL and AMAASDISLL), and papain yielded three (NEINQFYQK, FQSEEQQQTEDELQDK, and AMAASDISL), whereas no matching peptides were detected in the pepsin hydrolysate.

Collectively, these results indicated that enzymatic hydrolysis effectively targeted cleavage sites within the predicted cross-reactive epitope regions, as evidenced by the MS-identified epitope-overlapping peptides. The concomitant significant reduction in antibody binding by ELISA further corroborated that this targeted cleavage contributed to the decreased immunoreactivity, supporting the potential of these enzymatic treatments for reducing cross-reactivity.

## Figures and Tables

**Figure 10:**
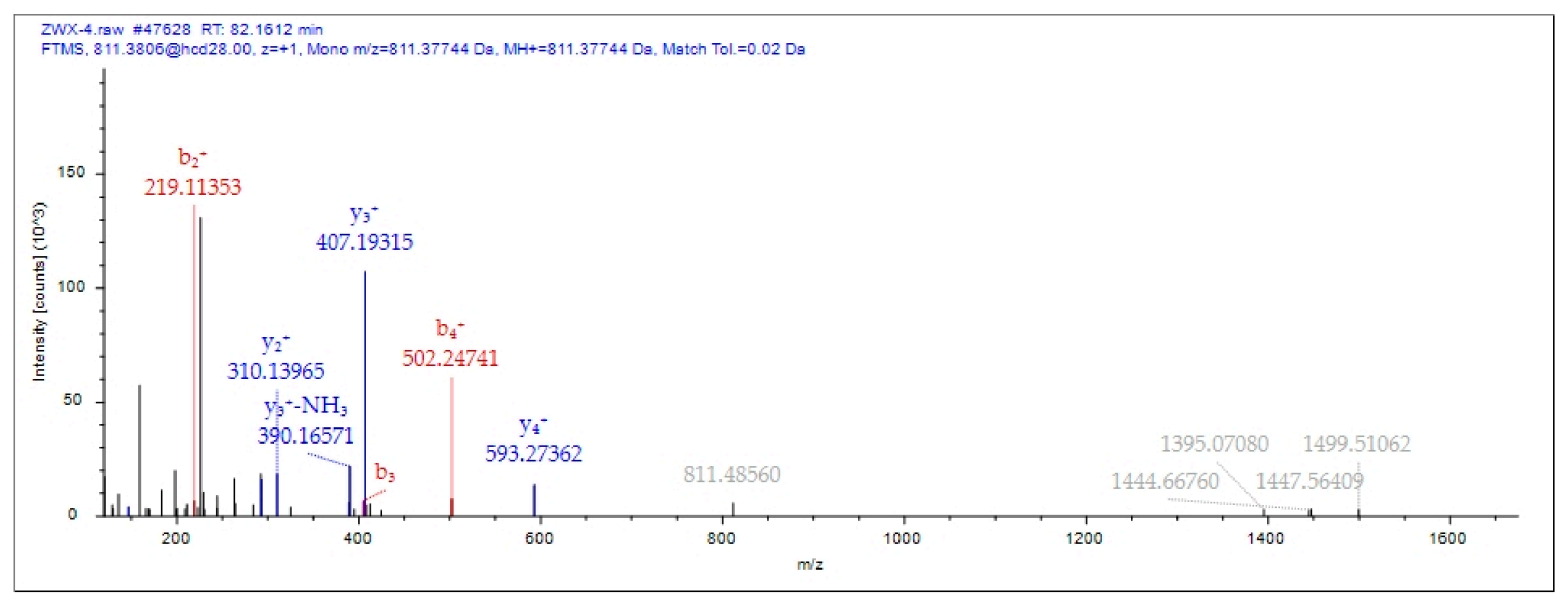
Mass spectrum of FAWPQY (177–182).

**Figure 11:**
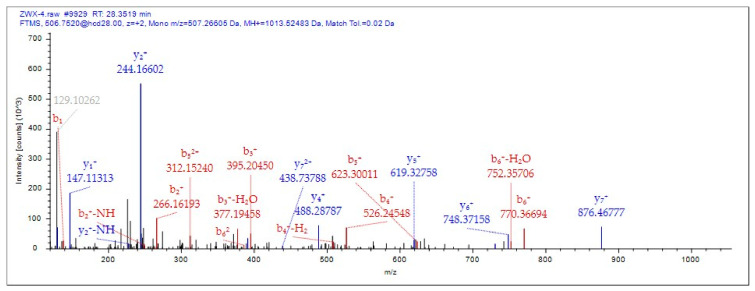
Mass spectrum of LKDLKDY (12–18).

**Figure 12:**
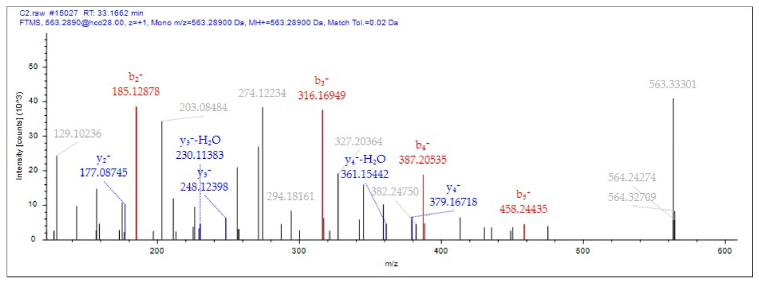
Mass spectrum of LAMAAS (22–27).

**Figure 13:**
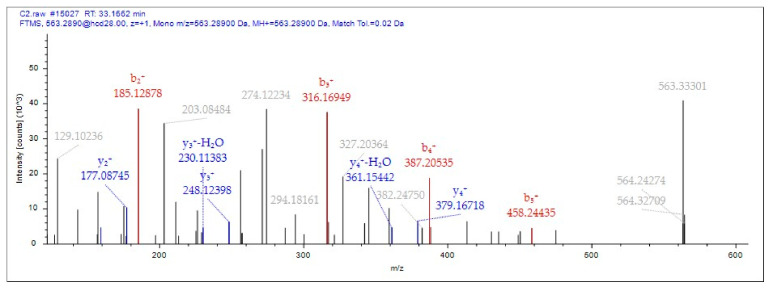
Mass spectrum of IPIQYVLS (26–33).

**Figure 14:**
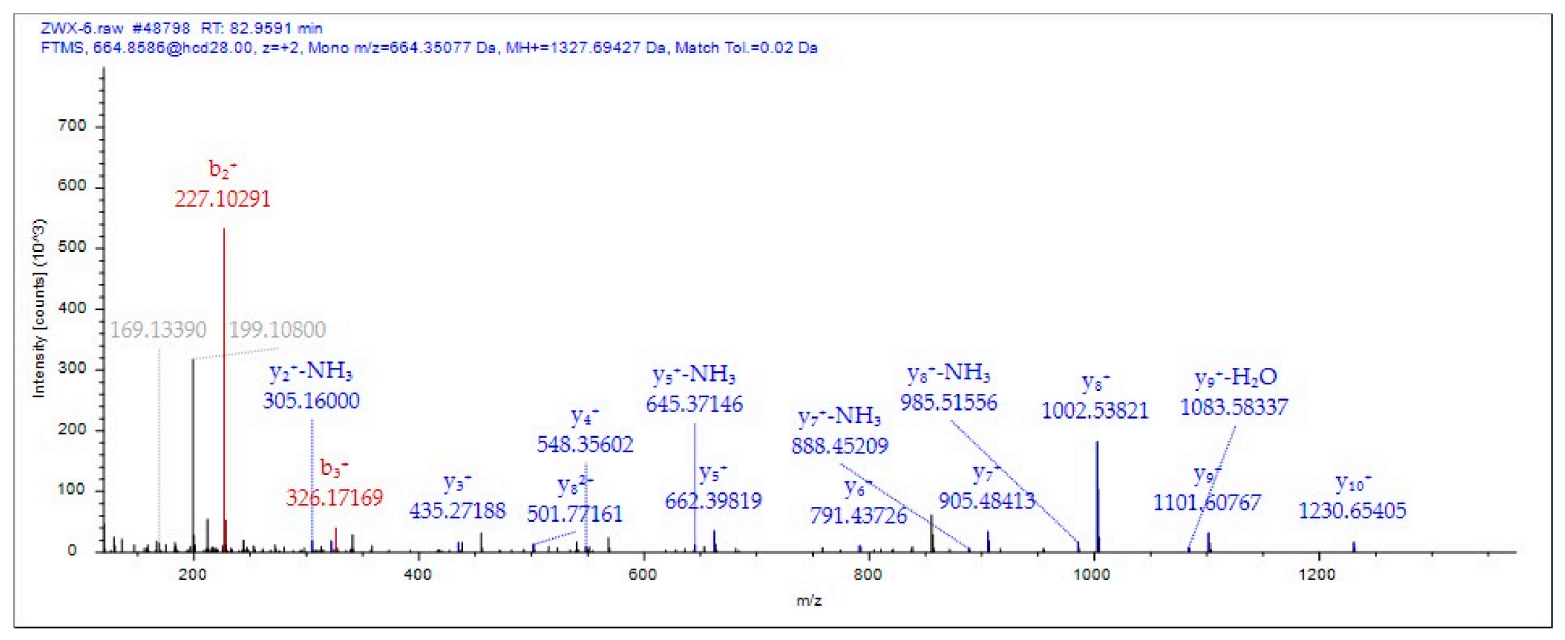
Mass spectrum of PPQSVLS (177–183).

**Figure 15:**
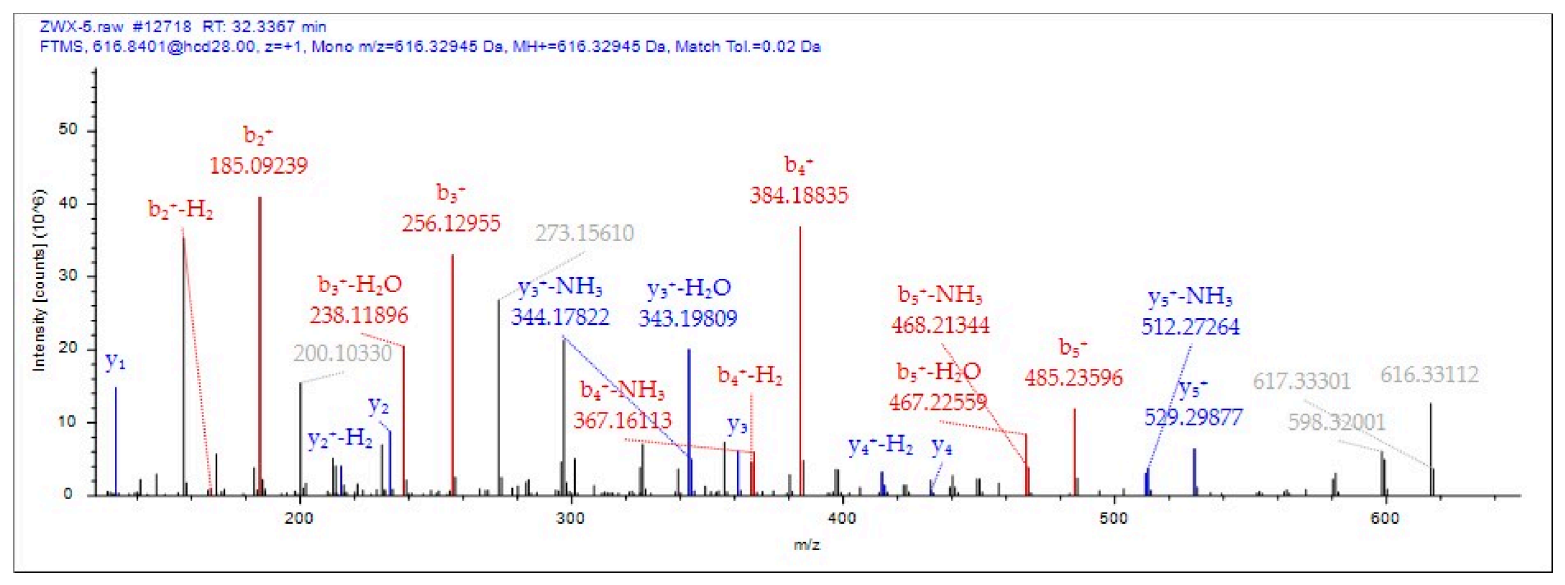
Mass spectrum of LPYPYY (84–89).

**Figure 16:**
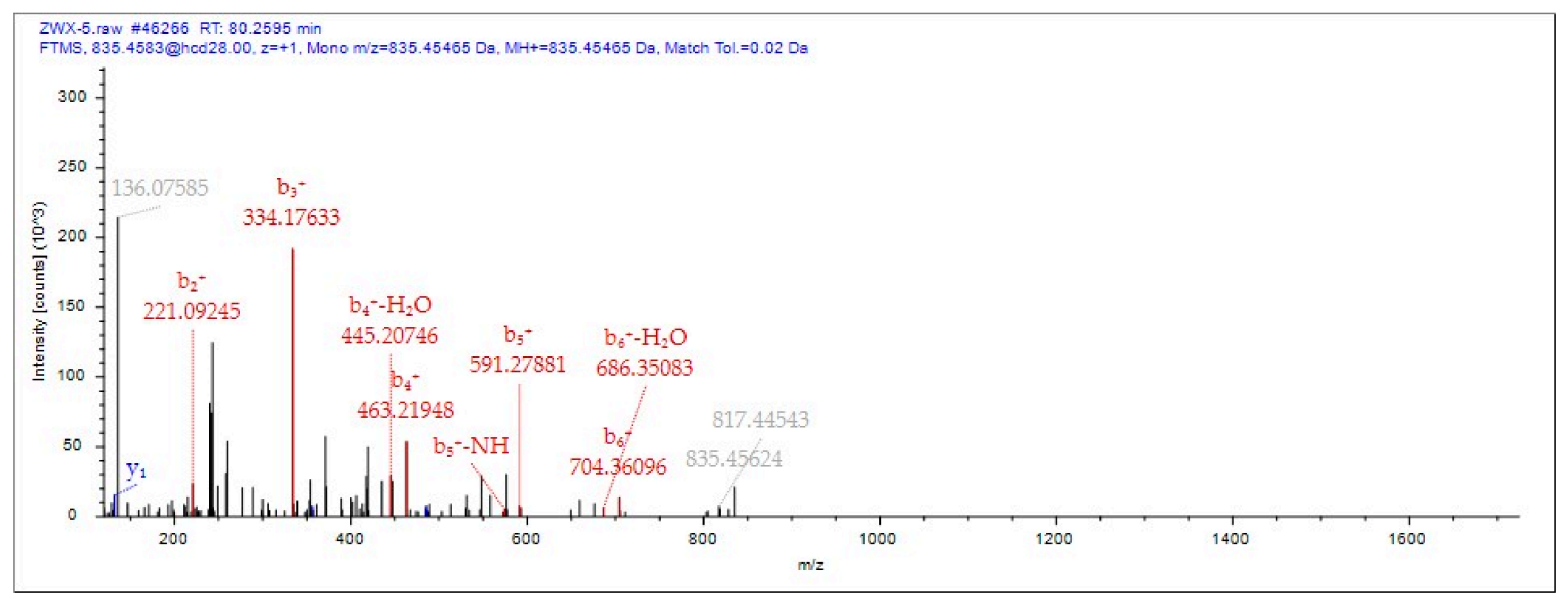
Mass spectrum of YLGYLEQL (91–98).

**Figure 17:**
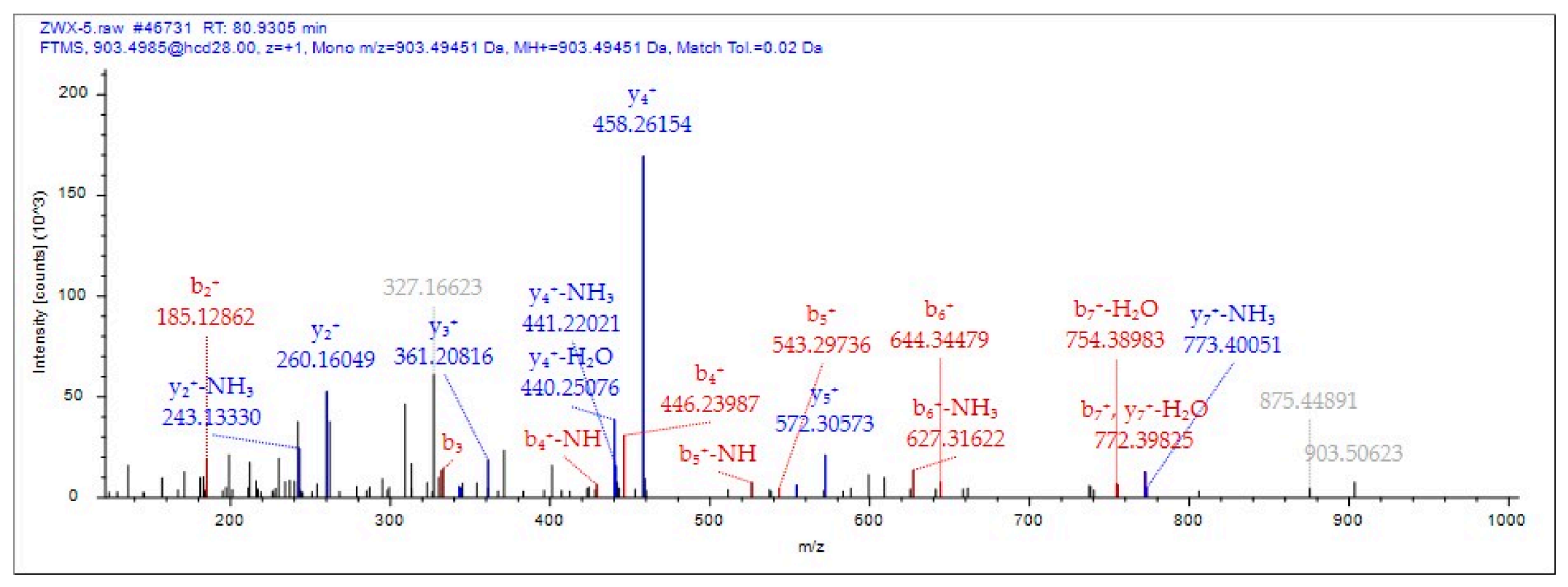
Mass spectrum of AMAASDISL (23–32).

**Figure 18:**
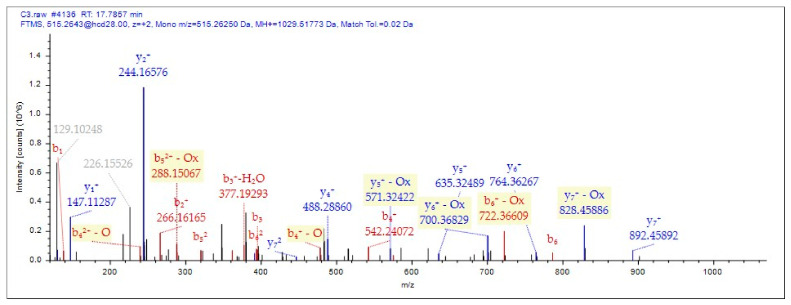
Mass spectrum of NEINQFYQK (84–92).

**Figure 19:**
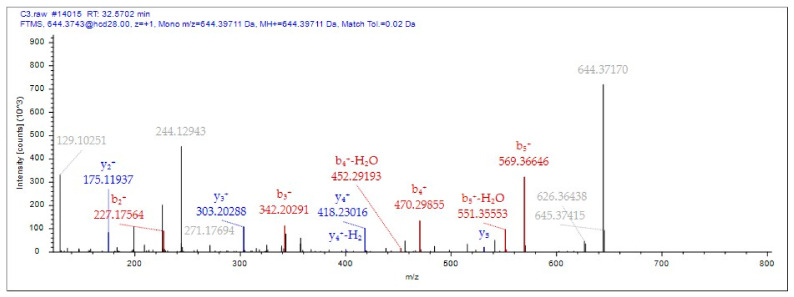
Mass spectrum of FQSEEQQQTEDELQDK (33–48).

**Figure 20:**
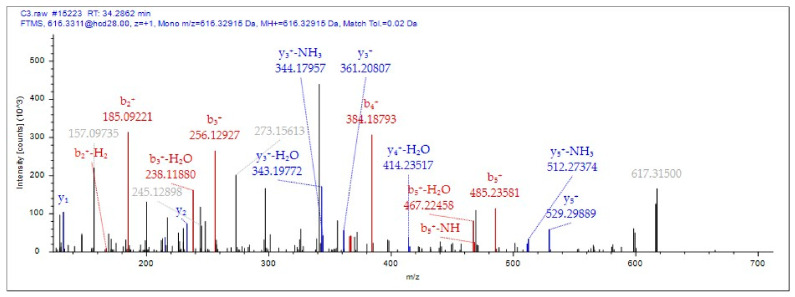
Mass spectrum of AMAASDISL (21–31).

**Table 1:** Prediction of similar sequences between cow milk and goat milk.

	**Cow Milk Protein**	**Goat Milk Protein**	**Score**	**Expect**	**Identities**	**Positives**	**Gaps**
α S1-casein	NELSKDIGSESTEDQAMED (53–71)	NELSKDIGSESTEDQAMED (53–71)	40.4 bits (93)	2 × 10^−11^	19/19 (100%)	19/19 (100%)	0/19 (0%)
	GAWYYVPLGTQYTDAPSFSD (177–196)	GAWYYLPLGTQYTDAPSFSD (177–196)	48.1 bits (113)	5 × 10^−14^	19/20 (95%)	20/20 (100%)	0/20 (0%)
	VPSERYLGYLEQLLRLKKY (101–119)	VPSERYLGYLEQLLKLKKY (101–119)	39.3 bits (90)	7 × 10^−11^	18/19 (95%)	19/19 (100%)	0/19 (0%)
	SISSSEEIVPNSVEQKHIQK (79–98)	SSSSSEEIVPNSAEQKYIQK (79–98)	33.9 bits (76)	6 × 10^−9^	17/20 (85%)	18/20 (90%)	0/20 (0%)
	EIVPNSAEER (125–134)	EIVPNSAEQK (85–94)	20.8 bits (42)	2 × 10^−4^	8/10 (80%)	10/10 (100%)	0/10 (0%)
	EVLNENLLRFFVALFPEVF (29–47)	EVPNENLLRFVVAPFPEVF (29–47)	30.4 bits (67)	9 × 10^−8^	16/19 (84%)	16/19 (84%)	0/19 (0%)
α S2-casein	QKALNEINQFYQKFPQYLQY (94–113)	QKALNEINQFYQKFPQYLQY (95–114)	43.5 bits (101)	2 × 10^−12^	20/20 (100%)	20/20 (100%)	0/20 (0%)
	LKKISQRYQKFALPQYLK (179–196)	LNEINQFYQKF--PQYLQ (98–113)	20.8 bits (42)	3 × 10^−4^	11/18 (61%)	14/18 (77%)	2/18 (11%)
	REQLSTSEENSKKTVDMEST (140–159)	REQLSTSEENSKKTIDMEST (141–160)	40.0 bits (92)	3 × 10^−11^	19/20 (95%)	20/20 (100%)	0/20 (0%)
	KNRLNFLKKISQRYQKFALP (173–192)	KNRLNFLKIISQYYQKFAWP (174–193)	36.2 bits (82)	9 × 10^−10^	17/20 (85%)	17/20 (85%)	0/20 (0%)
	LNEINQFYQKF (97–107)	LKIISQYYQKF (180–190)	17.3 bits (33)	4 × 10^−3^	7/11 (64%)	9/11 (81%)	0/11 (0%)
	QHQKAMKPWIQPKTKVIPYV (200–219)	QHQKAMKRWTQPKTNAIPYV (201–220)	37.0 bits (84)	4 × 10^−10^	16/20 (80%)	16/20 (80%)	0/20 (0%)
	WDQVKRNAVP (124–133)	WTQPKTNAIP (209–218)	18.1 bits (35)	3 × 10^−3^	6/10 (60%)	7/10 (70%)	0/10 (0%)
	AKNTMEHVSSSEESI (15–29)	AKHKMEHVSSSEEPI (15–29)	25.4 bits (54)	6 × 10^−6^	12/15 (80%)	13/15 (86%)	0/15 (0%)
β-casein	KFQSEEQQQTEDELQDKIHP (193–212)	KFQSEEQQQTEDELQDKIHP (47–66)	42.7 bits (99)	8 × 10^−12^	20/20 (100%)	20/20 (100%)	0/20 (0%)
	PTVMFPPQSVLSLSQSKVL (314–332)	PTVMFPPQSVLSLSQPKVL (167–186)	36.2 bits (82)	2 × 10^−9^	18/19 (95%)	18/19 (94%)	0/19 (0%)
	EELNVPGEIVES (165–176)	EELNVVGETVES (19–30)	16.9 bits (32)	1.1 × 10^−3^	10/12 (83%)	10/12 (83%)	0/12 (0%)
	VKEAMAPKHKEMPFPKYPVE (259–278)	VKETMVPKHKEMPFPKYPVE (113–32)	40.4 bits (93)	5 × 10^−11^	18/20 (90%)	18/20 (90%)	0/20 (0%)
κ-casein	FSDKIAKYIPIQYVLSRYPS (39–58)	FDDKIAKYIPIQYVLSRYPS (39–58)	42.4 bits (98)	4 × 10^−12^	19/20 (95%)	19/20 (95%)	0/20 (0%)
	QDKTEIPTINTIASGEPT (135–152)	QDKTEVPAINTIASAEPT (135–152)	34.3 bits (77)	3 × 10^−9^	15/18 (83%)	16/18 (88%)	0/18 (0%)
	NQFLPYPYYAKPAAVRSPAQ (74–93)	NQFLPYPYYAKPVAVRSPAQ (74–93)	42.4 bits (98)	5 × 10^−12^	19/20 (95%)	19/20 (95%)	0/20 (0%)
α -lactalbumin	KILDKVGINYWLAHKALCSE (113–132)	KILDKVGINYWLAHKALCSE (113–132)	44.7 bits (104)	3 × 10^−13^	20/20 (100%)	20/20 (100%)	0/20 (0%)
	GILFHATQAEQLTKCEVFRE (11–30)	GILFHATQAEQLTKCEVFQK (11–30)	41.6 bits (96)	4 × 10^−12^	18/20 (90%)	20/20 (100%)	0/20 (0%)
β-lactoglobulin	YSLAMAASDISLLDAQSAPL (36–55)	YSLAMAASDISLLDAQSAPL (38–57)	39.7 bits (91)	3 × 10^−11^	20/20 (100%)	20/20 (100%)	0/20 (0%)
	LNENKVLVLDTDYKKYLLVC (103–122)	LNENKVLVLDTDYKKYLLFC (105–124)	40.8 bits (94)	1 × 10^−11^	19/20 (95%)	19/20 (95%)	0/20 (0%)
	CGAQALIVTQTMKGLDIQK (12–30)	CGIQAIIVTQTMKGLDIQK (14–32)	36.6 bits (83)	4 × 10^−10^	17/19 (89%)	18/19 (94%)	0/19 (0%)
	PEQSLVCQCLVRTPEVDDEA (129–148)	PEQSLACQCLVRTPEVDKEA (131–150)	40.4 bits (93)	2 × 10^−11^	18/20 (90%)	18/20 (90%)	0/20 (0%)
	LPMHIRLSFNPTQLEEQCHI (159–178)	LPMHIRLAFNPTQLEGQCHV (161–180)	44.3 bits (103)	7 × 10^−13^	17/20 (85%)	19/20 (95%)	0/20 (0%)

**Table 2:** Prediction of similar sequences between cow milk and sheep milk.

	**Cow Milk Protein**	**Sheep Milk Protein**	**Score**	**Expect**	**Identities**	**Positives**	**Gaps**
α S1-casein	GAWYYVPLGTQYTDAPSFSD (177–196)	GAWYYLPLGTQYTDAPSFSD (169–188)	48.1 bits (113)	5 × 10^−14^	19/20 (95%)	20/20 (100%)	0/20 (0%)
	VPSERYLGYLEQLLRLKKY (101–119)	VPSERYLGYLEQLLRLKKY (101–119)	40.8 bits (94)	2 × 10^−11^	19/19 (100%)	19/19 (100%)	0/19 (0%)
	NELSKDIGSESTEDQAMED (53–71)	NELSKDIGSESIEDQAMED (53–71)	38.1 bits (87)	2 × 10^−10^	18/19 (95%)	18/19 (94%)	0/19 (0%)
	SISSSEEIVPNSVEQKHIQK (79–98)	SSSSSEEIVPNSAEQKYIQK (79–98)	33.9 bits (76)	6 × 10^−9^	17/20 (85%)	18/20 (90%)	0/20 (0%)
	EIVPNSAEER (125–134)	EIVPNSAEQK (85–94)	20.8 bits (42)	2 × 10^−4^	8/10 (80%)	10/10 (100%)	0/10 (0%)
α S2-casein	LNEINQFYQKFPQYLQYLYQ (97–116)	LNEINQFYQKFPQYLQYLYQ (98–117)	43.5 bits (101)	2 × 10^−12^	20/20 (100%)	20/20 (100%)	0/20 (0%)
	LKKISQRYQKFALPQYLKTVYQ (179–200)	LNEINQFYQKF--PQYLQYLYQ (98–117)	24.6 bits (52)	1 × 10^−5^	13/22 (59%)	17/22 (77%)	2/22 (9%)
	PSKENLCSTFCKEVVRNANE (45–64)	PRKEKLCTTSCEEVVRNADE (46–65)	33.5 bits (75)	8 × 10^−9^	14/20 (70%)	17/20 (85%)	0/20 (0%)
	REQLSTSEENSKKTVDMEST (140–159)	REQLSTSEENSKKTIDMEST (141–160)	40.0 bits (92)	3 × 10^−11^	19/20 (95%)	20/20 (100%)	0/20 (0%)
	LAKNTMEHVSSSEESI-ISQ (14–32)	LAKHKMEHVSSSEEPINISQ (14–33)	27.7 bits (60)	9 × 10^−7^	16/20 (80%)	17/20 (85%)	1/20 (5%)
	KNRLNFLKKISQRYQKFALP (173–192)	KNRLNFLKKISQYYQKFAWP (174–193)	38.5 bits (88)	1 × 10^−10^	18/20 (90%)	18/20 (90%)	0/20 (0%)
	LNEINQFYQKF (97–107)	LKKISQYYQKF (180–190)	18.9 bits (37)	1 × 10^−3^	7/11 (64%)	10/11 (90%)	0/11 (0%)
	QHQKAMKPWIQPKTKVIPY (200–218)	QHQKAMKPWTQPKTNAIPY (201–219)	39.3 bits (90)	7 × 10^−11^	16/19 (84%)	16/19 (84%)	0/19 (0%)
	VLNPWDQVKRNAVP (120–133)	AMKPWTQPKTNAIP (205–217)	21.6 bits (44)	1 × 10^−4^	7/14 (50%)	9/14 (64%)	0/14 (0%)
β-casein	KFQSEEQQQTEDELQDKIHP (47–66)	KFQSEEQQQTEDELQDKIHP (46–65)	42.7 bits (99)	4 × 10^−12^	20/20 (100%)	20/20 (100%)	0/20 (0%)
	TVMFPPQSVLSLSQSKVLPV (169–188)	TVMFPPQSVLSLSQPKVLPV (168–187)	36.2 bits (82)	8 × 10^−10^	19/20 (95%)	19/20 (95%)	0/20 (0%)
	EELNVPGEIVES (19–30)	EELNVVGETVES (18–29)	17.3 bits (33)	4 × 10^−3^	10/12 (83%)	10/12 (83%)	0/12 (0%)
	VKEAMAPKHKEMPFPKYPVE (113–132)	VKETMVPKHKEMPFPKYPVE (112–131)	39.7 bits (91)	5 × 10^−11^	18/20 (90%)	18/20 (90%)	0/20 (0%)
κ-casein	FSDKIAKYIPIQYVLSRYPS (39–58)	FDDKIAKYIPIQYVLSRYPS (39–58)	42.4 bits (98)	4 × 10^−12^	19/20 (95%)	19/20 (95%)	0/20 (0%)
	QDKTEIPTINTIASGEPT (135−152)	QDKTEIPAINTIASAEPT (135–152)	35.0 bits (79)	2 × 10^−9^	16/18 (89%)	16/18 (88%)	0/18 (0%)
	EDSPEVIESPPEINTVQVT (168–186)	EASSESIASAPETNTAQVT (170–188)	23.5 bits (49)	2 × 10^−5^	12/19 (63%)	12/19 (63%)	0/19 (0%)
	NQFLPYPYYAKPAAVRSPAQ (74–93)	NQFLPYPYYAKPVAVRSPAQ (74–93)	42.4 bits (98)	5 × 10^−12^	19/20 (95%)	19/20 (95%)	0/20 (0%)
α -lactalbumin	KILDKVGINYWLAHKALCS (113–131)	KILDKVGINYWLAHKALCS (113–131)	42.4 bits (98)	2 × 10^−12^	19/19 (100%)	19/19 (100%)	0/19 (0%)
	GILFHATQAEQLTKCEVFRE (11–30)	GILFHATQAEQLTKCEAFQK (11–30)	40.0 bits (92)	1 × 10^−11^	17/20 (85%)	19/20 (95%)	0/20 (0%)
β-lactoglobulin	LNENKVLVLDTDYKKYLLVC (103–122)	LNENKVLVLDTDYKKYLLFC (105–124)	41.2 bits (95)	6 × 10^−12^	19/20 (95%)	19/20 (95%)	0/20 (0%)
	YSLAMAASDISLLDAQSAPL (36–55)	HSLAMAASDISLLDAQSAPL (38–57)	35.4 bits (80)	7 × 10^−10^	19/20 (95%)	20/20 (100%)	0/20 (0%)
	CGAQALIVTQTMKGLDIQK (12–30)	CGVQAIIVTQTMKGLDIQK (14–32)	38.5 bits (88)	5 × 10^−11^	17/19 (89%)	18/19 (94%)	0/19 (0%)
	PEQSLVCQCLVR (129–140)	PEQSLACQCLVR (131–142)	27.7 bits (60)	6 × 10^−7^	11/12 (92%)	11/12 (91%)	0/12 (0%)

**Table 3:** Content of the 20 amino acids within goat milk protein.

**Amino Acid**	**Molecular**	**Concentration μmol/L**
Ser	105.09	116
Gly	75.07	51.6
His	155.15	38.8
Thr	119.13	84.8
Gln	146.15	237
Asp	133.1	115
Ala	89.09	97.8
Arg	174.2	45.4
Pro	115.14	249
Cys	121.16	13.3
Lys	146.19	152
Met	149.2	47
Val	117.15	142
Tyr	181.19	45.3
Ile	131.18	79.8
Leu	131.17	163
Phe	165.19	66.5

**Table 4:** Free amino acid content in goat milk protein.

**OD**	3.018
**NH_2_ Concentration (mg/mL)**	7.689

**Table 5:** The amino acid sequence alignment of peptides of hydrolysates was analyzed by mass spectrometry.

Protease	Allergen	Mass Spectrometry Sequence	Similar Sequence	Overlapping Sequence
Bromelain	α_S2_-casein	FAWPQY (177–182)	KNRLNFLKIISQYYQKFAWP (173–192)	FAWPQY (177–182)
	α-lactalbumin	LKDLKDY (12–18)	GILFHATQAELTKCEVFQK (11–30)	LKDLKDY (12–18)
Protamex	β-lactoglobulin	LAMAAS (22–27)	YSLAMAASDISLLDAQSAPL (36–55)	LAMAAS (22–27)
Alcalase	β-casein	PPQSVLS (177–183	PTVMPPPQSVLSLSQKVL (314–332)	PPQSVLS (177–183)
	κ-casein	IPIQYVLS (26–33)	FSDKIAKYIPIQYVLSRYPS (39–58)	IPIQYVLS (26–33)
		LPYPYY (84–89)	NOFLPYPYYAKPAAVRSPAQ (74–93)	LPYPYY (84–89)
Papain(s)	α_S2_-casein	NEINQFYQK (84–92)	QKALNEINQFYQKFQYLQY (94–113)	NEINQFYQK (84–92)
	β-casein	FQSEEQQQTEDELQDK (33–48)	KFQSEEQQQTEDELQDKIHP (193–212)	FQSEEQQQTEDELQDK (33–48)
	β-lactoglobulin	AMAASDISL (21–31)	YSLAMAASDISLLDAQSAPL (36–55)	AMAASDISL (21–31)
Trypsin	α_S1_-casein	YLGYLEQL (91–98)	VPSERYLGYLEQLLRLKKY (101–119)	YLGYLEQL (91–98)
	β-lactoglobulin	AMAASDISLL (23–32)	YSLAMAASDISLLDAQSAPL (36–55)	AMAASDISLL (23–32)

**Table 6:** Optimal conditions for the hydrolysis of goat milk protein by various proteases.

Protease	Concentration (%)	pH	Temperature
Papain (s)	3	7.0	60 °C
Alcalase	3	8.0	60 °C
Pepsin	3	2.0	37 °C
Trypsin	3	8.0	50 °C
Bromelain	3	7.0	50 °C

**Table 7:** Protease hydrolysis sites.

Protease	Hydrolysis Sites
Protamex	S
Alcalase	S, G, Y, F, W
Bromelain	Lys, Ala, Tyr, Gly
Pepsin	Phe, Leu
Papain (s)	L, G, K, R
Trypsin	R, P, L

## Data Availability

The original contributions presented in this study are included in the article. Further inquiries can be directed to the corresponding author.

## Abbreviations

The following abbreviations are used in this manuscript:

CMA	Cow’s Milk Allergy
BLAST	Basic Local Alignment Search Tool
BLASTP	protein–protein BLAST
SDS-PAGE	sodium dodecyl sulfate-polyacrylamide gel electrophoresis
TMB	3,3′,5,5′-tetramethylbenzidine
ELISA	enzyme-linked immunosorbent assay
BSA	bovine serum albumin
PBST	Phosphate Buffered Saline with Tween-20
HRP	Horseradish Peroxidase
IgE	Immunoglobulin E
IgG	Immunoglobulin G

## References

[1] Ji Z., Dong R., Du Q., Jiang H., Fan R., Bu D., Wang J., Yu Z., Han R., Yang Y. (2024). Insight into differences in whey proteome from human and eight dairy animal species for formula humanization. Food Chem..

[2] Villa C., Costa J., Oliveira M.B.P.P., Mafra I. (2018). Bovine milk allergens: A comprehensive review. Compr. Rev. Food Sci. Food Saf..

[3] Maryniak N.Z., Sancho A.I., Hansen E.B., Bøgh K.L. (2022). Alternatives to Cow’s Milk-Based Infant Formulas in the Prevention and Management of Cow’s Milk Allergy. Foods.

[4] Fox A., Bird J.A., Fiocchi A., Knol J., Meyer R., Salminen S., Sitang G., Szajewska H., Papadopoulos N. (2019). The potential for pre-, pro-and synbiotics in the management of infants at risk of cow’s milk allergy or with cow’s milk allergy. World Allergy Organ. J..

[5] Pessato T.B., Carvalho N.C.D., Tavano O.L., Fernandes L.G.R., Zollner R.d.L., Netto F.M. (2016). Whey protein isolate hydrolysates obtained with free and immobilized Alcalase: Characterization and detection of residual allergens. Food Res. Int..

[6] Chen Q., Yin Q., Xie Q., Liu S., Guo Z., Li B. (2023). Elucidating gut microbiota and metabolite patterns shaped by goat milk-based infant formula feeding in mice colonized by healthy infant feces. Food Chem..

[7] Jenness R. (1980). Composition and characteristics of goat milk: Review 1968–1979. J. Dairy Sci..

[8] International Dairy Federation (2011). Milk of Sheep, Goat, and Buffalo: A Public Health Review.

[9] Bevilacqua C., Martin P., Candalh C., Fauquant J., Piot M., Roucayrol A.-M., Pilla F., Heyman M. (2001). Goats’ milk of defective alpha(s1)-casein genotype decreases intestinal and systemic sensitization to beta-lactoglobulin in guinea pigs. J. Dairy Res..

[10] Zhang M., Deng Z., Song H., Zhao C., Zou Y., Li Y., Zheng L. (2025). Whey protein and endogenous oligopeptide profiles: A comparative study of human and major domestic animal milk. Food Biosci..

[11] Albenzio M., Santillo A., Ciliberti M.G., Figliola L., Caroprese M., Polito A., Messina G. (2018). Milk nutrition and childhood epilepsy: An ex vivo study on cytokines and oxidative stress in response to milk protein fractions. J. Dairy Sci..

[12] Albenzio M., Santillo A., Avondo M., Nudda A., Chessa S., Pirisi A., Banni S. (2016). Nutritional properties of small ruminant food products and their role on human health. Small Rumin. Res..

[13] Lajnaf R., Feki S., Ben Ameur S., Attia H., Kammoun T., Ayadi M.A., Masmoudi H. (2023). Recent advances in selective allergies to mammalian milk proteins not associated with Cow’s Milk Proteins Allergy. Food Chem. Toxicol..

[14] Schade R.P., Van Ieperen-Van Dijk A.G., Van Reijsen F.C., Versluis C., Kimpen J.L.L., Knol E.F., Bruijnzeel-Koomen C.A.F.M., Van Hoffen E. (2000). Differences in antigen-specific T-cell responses between infants with atopic dermatitis with and without cow’s milk allergy: Relevance of TH2 cytokines. J. Allergy Clin. Immunol..

[15] Saarela T., Kokkonen J., Koivisto M. (2005). Macronutrient and energy contents of human milk fractions during the first six months of lactation. Acta Paediatr..

[16] Benjamin-van Aalst O., Dupont C., van der Zee L., Garssen J., Knipping K. (2024). Goat Milk Allergy and a Potential Role for Goat Milk in Cow’s Milk Allergy. Nutrients.

[17] Wija A.P., Sari D.P., Prasetyo D. (2023). Cow’s milk alternatives for children with cow’s milk protein allergy—Review of health benefits and risks of allergic reaction. Kesans J. Health Sci..

[18] Bernard H., Ah-Leung S., Tilleul S., Drumare M.-F., Paty E., Bidat E., Wal J.-M., Hazebrouck S. (2012). Specificity of IgE antibodies from patients allergic to goat’s milk and tolerant to cow’s milk determined with plasmin-derived peptides of bovine and caprine β-caseins. Mol. Nutr. Food Res..

[19] Facioni M.S., Raspini B., Pivari F., Dogliotti E., Cena H. (2020). Nutritional management of lactose intolerance: The importance of diet and food labelling. J. Transl. Med..

[20] Hodgkinson A.J., Mcdonald N.A., Kivits L.J., Hurford D., Fahey S., Prosser C. (2012). Allergic responses induced by goat milk αs_1_-casein in a murine model of gastrointestinal atopy. J. Dairy Sci..

[21] Yao B., Zhang L., Liang S., Zhang C. (2012). SVMTriP: A method to predict antigenic epitopes using support vector machine to integrate tri-peptide similarity and propensity. PLoS ONE.

[22] Herman R., Song P., Mirsky H.P., Roper J.M. (2021). Evidence-based regulations for bioinformatic prediction of allergen cross-reactivity are needed. Regul. Toxicol. Pharmacol..

[23] Bellioni-Businco B., Paganelli R., Lucenti P., Giampietro P.G., Perbornc H., Businco L. (1999). Allergenicity of goat’s milk in children with cow’s milk allergy. J. Allergy Clin. Immunol..

[24] Hazebrouck S., Ah-Leung S., Bidat E., Paty E., Drumare M., Tilleul S., Adel-Patient K., Wal J., Bernard H. (2014). Goat’s milk allergy without cow’s milk allergy: Suppression of non-cross-reactive epitopes on caprine β-casein. Clin. Exp. Allergy.

[25] Restani P., Beretta B., Fiocchi A., Ballabio C., Galli C.L. (2002). Cross-reactivity between mammalian proteins. Ann. Allergy Asthma Immunol..

[26] Järvinen K.M., Beyer K., Vila L., Chatchatee P., Busse P.J., Sampson H.A. (2002). B-cell epitopes as a screening instrument for persistent cow’s milk allergy. J. Allergy Clin. Immunol..

[27] Lisson M., Novak N., Erhardt G. (2014). Immunoglobulin E epitope mapping by microarray immunoassay reveals differences in immune response to genetic variants of caseins from different ruminant species. J. Dairy Sci..

[28] Maynard F., Chatel J.M., Wal J.M. (1999). Immunological IgE cross-reactions of bovine and human α-lactalbumins in cow’s milk allergic patients. Food Agric. Immunol..

[29] Hu W., Xie F., Wu Y., Men X., Yang A., Wu Z., Gao J., Li X., Chen H. (2025). Identification and Validation of Key Amino Acids in IgE Linear Epitopes of β-Lactoglobulin: Comparison of Recognition Patterns of Chinese Bovine Milk-Allergic Sera with Different Symptoms. J. Agric. Food Chem..

[30] Meulenbroek L.A.P.M., Oliveira S., Den Hartog Jager C.F., Klemans R.J.B., Lebens A.F.M., van Baalen T., Knulst A.C., Bruijnzeel-Koomen C.A.F.M., Garssen J., Knippels L.M.J. (2014). The degree of whey hydrolysis does not uniformly affect in vitro basophil and T cell responses of cow’s milk-allergic patients. Clin. Exp. Allergy.

[31] Bu G., Luo Y., Chen F., Liu K., Zhu T. (2013). Milk processing as a tool to reduce cow’s milk allergenicity: Amini-review. Dairy Sci. Technol..

[32] Hahn J., Yun S.-S., Lee W.-J., Kim J.-W., Ha H.-K., Yoo M., Hwang H.-J., Jeon W.-M., Han K.-S. (2016). Hydrolysis by Alcalase improves hypoallergenic properties of goat milk protein. Korean J. Food Sci. Anim. Resour..

[33] Li H.B., Gao Q., Gao X., Long G., Bian M., Feng S., Mu S., Li H., Yu J. (2025). Integration LC-MS/MS and molecular modeling techniques to elucidate the molecular mechanisms of goat whey protein-IgG interactions. Food Chem..

[34] Li H., Zhao Y., Jiang Y., Li J., Yang J., Xue Y., Kang Z., Zulewska J., Li H., Yu J. (2023). Extensive hydrolysis of milk protein and its peptidomic antigenicity analysis by LC-MS/MS. J. Food Sci..

[35] Mari A., Scala E., Palazzo P., Ridolfi S., Zennaro D., Carabella G. (2006). Bioinformatics applied to allergy: Allergen databases, from collecting sequence information to data integration. The Allergome platform as a model. Cell. Immunol..

[36] Sayers E.W., Beck J., Bolton E.E., Bourexis D., Brister J.R., Canese K., Comeau D.C., Funk K., Kim S., Klimke W. (2021). Database resources of the National Center for Biotechnology Information. Nucleic Acids Res..

[37] Altschul S.F., Madden T.L., Schäffer A.A., Zhang J., Zhang Z., Miller W., Lipman D.J. (1997). Gapped BLAST and PSI-BLAST: A new generation of protein database search programs. Nucleic Acids Res..

[38] Fu Y., Liu J., Hansen E.T., Bredie W.L., Lametsch R. (2018). Structural characteristics of low bitter and high umami protein hydrolysates prepared from bovine muscle and porcine plasma. Food Chem..

[39] Yolandani, Ma H., Liu D., Wang X., Liu X., Zhang X., Sun X., Li D., Zhou D., Li Y. (2024). Comparison of prediction models for soy protein isolate hydrolysates bitterness built using sensory, spectrofluorometric and chromatographic data from varying enzymes and degree of hydrolysis. Food Chem..

[40] (2016). National Food Safety Standard—Determination of Amino Acid in Foods.

[41] Docena G., Rozenfeld P., Fernández R., Fossati C.A. (2002). Evaluation of the residual antigenicity and allergenicity of cow‘s milk substitutes by in vitro tests. Allergy.

[42] Cui Q., Zhang Z., Li M., Zhou M., Sun X. (2023). Peptide profiles and allergy-reactivity of extensive hydrolysates of milk protein. Food Chem..

